# Lack of SPNS1 results in accumulation of lysolipids and lysosomal storage disease in mouse models

**DOI:** 10.1172/jci.insight.175462

**Published:** 2024-03-07

**Authors:** Hoa T.T. Ha, SiYi Liu, Xuan T.A. Nguyen, Linh K. Vo, Nancy C.P. Leong, Dat T. Nguyen, Shivaranjani Balamurugan, Pei Yen Lim, YaJun Wu, Eunju Seong, Toan Q. Nguyen, Jeongah Oh, Markus R. Wenk, Amaury Cazenave-Gassiot, Zuhal Yapici, Wei-Yi Ong, Margit Burmeister, Long N. Nguyen

**Affiliations:** 1 Department of Biochemistry, Yong Loo Lin School of Medicine,; 2 Singapore Lipidomics Incubator (SLING), Life Sciences Institute, and; 3 Department of Anatomy, Yong Loo-Lin School of Medicine, National University of Singapore, Singapore.; 4 Michigan Neuroscience Institute, University of Michigan, Ann Arbor, USA.; 5 Department of Neurology, Faculty of Medicine, Istanbul University, Istanbul, Turkey.; 6 Departments of Computational Medicine and Biochemistry, Psychiatry, and Human Genetics, University of Michigan, Ann Arbor, USA.; 7 Cardiovascular Disease Research (CVD) Programme, Yong Loo Lin School of Medicine,; 8 Immunology Program, Life Sciences Institute, and; 9 Immunology Translational Research Program, Yong Loo Lin School of Medicine, National University of Singapore, Singapore.

**Keywords:** Aging, Metabolism, Autophagy, Embryonic development, Mouse models

## Abstract

Accumulation of sphingolipids, especially sphingosines, in the lysosomes is a key driver of several lysosomal storage diseases. The transport mechanism for sphingolipids from the lysosome remains unclear. Here, we identified SPNS1, which shares the highest homology to SPNS2, a sphingosine-1-phosphate (S1P) transporter, functions as a transporter for lysolipids from the lysosome. We generated *Spns1*-KO cells and mice and employed lipidomic and metabolomic approaches to reveal SPNS1 ligand identity. Global KO of *Spns1* caused embryonic lethality between E12.5 and E13.5 and an accumulation of sphingosine, lysophosphatidylcholines (LPC), and lysophosphatidylethanolamines (LPE) in the fetal livers. Similarly, metabolomic analysis of livers from postnatal *Spns1*-KO mice presented an accumulation of sphingosines and lysoglycerophospholipids including LPC and LPE. Subsequently, biochemical assays showed that SPNS1 is required for LPC and sphingosine release from lysosomes. The accumulation of these lysolipids in the lysosomes of *Spns1*-KO mice affected liver functions and altered the PI3K/AKT signaling pathway. Furthermore, we identified 3 human siblings with a homozygous variant in the *SPNS1* gene. These patients suffer from developmental delay, neurological impairment, intellectual disability, and cerebellar hypoplasia. These results reveal a critical role of SPNS1 as a promiscuous lysolipid transporter in the lysosomes and link its physiological functions with lysosomal storage diseases.

## Abstract 2

The deletion of lysolipid transporter SPNS1 in murine modles causes lipid accumulation, resulting in embryonic lethality and lysosomal storage disease.

## Graphical abstract

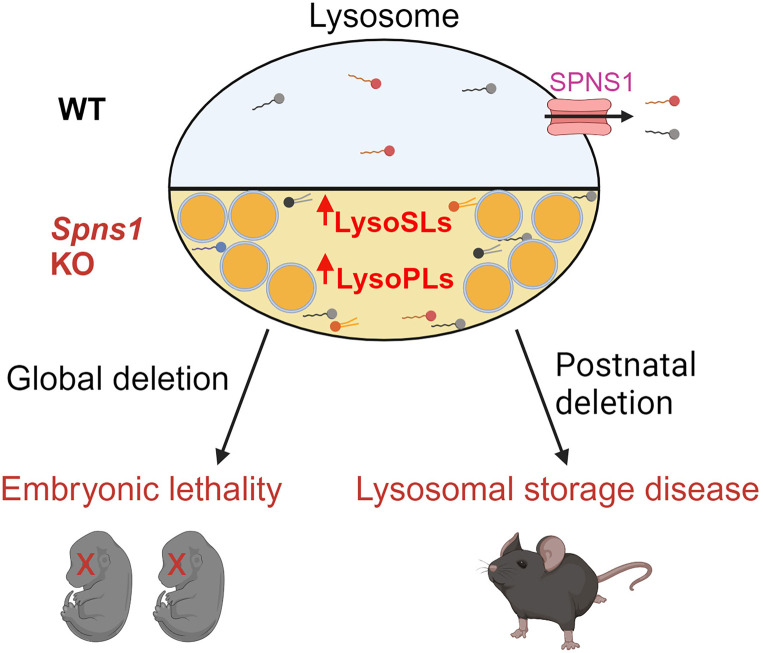

## Introduction

It is established that lysosomes are the hub for recycling sphingolipids, including sphingomyelins, ceramides, and glycosphingolipids, as well as glycerophospholipids such as phosphatidylcholines (PC) and phosphatidylethanolamines (PE). The recycling of sphingolipids appears particularly important for cellular functions because mutations of the enzymes and proteins involved in sphingolipid hydrolysis in the lysosome often result in pathological conditions, which are referred to as sphingolipid lysosomal storage diseases (SLSD) (1–3). Several types of SLSD described in the literature, such as Gaucher, Sandhoff, Fabry, Tay-Sachs, and Niemann-Pick type C diseases, remain untreatable (3, 4).

Complex glycerophospholipids and sphingolipids are delivered to the lysosomes via endocytosis or autophagic pathways, to be hydrolyzed by lysosomal enzymes (5). For glycerophospholipids, the hydrolysis of lipids to respective lysoglycerophospholipids such as lysophosphatidylcholines (LPC) and lysophosphatidylethanolamines (LPE) is thought to be mediated by a sole enzyme LPLA2 (6). LPC and LPE could be further hydrolyzed by uncharacterized enzymes to glycerophosphodiesters such as glycerophosphocholine (GPC) and glycerophosphoethanolamine (GPE) (7). The lysoglycerophospholipids can also be exported out of the lysosomes. With regards to sphingolipid hydrolysis in the lysosomes, this pathway has been relatively well characterized (8). One of the simplest by-products of this process is sphingosine, which does not undergo further degradation and must be released from the lysosome for recycling. In the acidic environment of the lysosomes, the amine group of sphingosines is protonated, preventing it from diffusing across the lysosomal membrane into the cytoplasm, where it can be converted into S1P or ceramides. We hypothesized that sphingosine species are transported out of the lysosomes via a protein-mediated process. Accumulation of these phospholipids, especially sphingosine species, has been shown to be detrimental to lysosomal homeostasis and functions (9, 10). However, the identity of the lysosomal transporter for sphingosines is unknown.

We identified Spinster protein homolog 1 (SPNS1), which belongs to a small family of 3 mammalian proteins, namely SPNS1–3. Human SPNS1 shares 57% identities with human SPNS2, which is a plasma membrane transporter for sphingosine-1-phosphate (S1P) (11–13). Several studies have shown that SPNS1 is expressed in the lysosomes and plays a role in the autophagy pathway (14). These reports helped us hypothesize that SPNS1 plays a role in the transport of sphingosines from the lysosome. In this study, we generated the KOs for *Spns1* in cell lines and mice. Here, we show that lack of SPNS1 resulted in the accumulation of sphingolipids such as sphingosines in mouse tissues and cell lines. Additionally, comprehensive metabolomics revealed that lack of SPNS1 also caused the accumulation of lysoglycerophospholipids such as LPC, LPE, lysophosphatidylinositols (LPI), lysophosphatidylglycerols (LPG), and lysoplasmalogens. We found that SPNS1 is responsible for the release of sphingosines and lysoglycerophospholipids such as LPC from the lysosomes. Global KO of *Spns1* in mice resulted in embryonic lethality with severe developmental defects. Furthermore, postnatal KO of *Spns1* in mice also caused the accumulation of these lysolipids in the lysosomes, with pathological conditions resembling SLSD. In the mouse liver, the accumulation of lysolipids in the lysosomes due to *Spns1* deletion affected multiple biological pathways, including PI3K/ATK signaling. The identification of SPNS1 as a lysolipid transporter lays a foundation for future investigations on the transport mechanism of lysolipids from the lysosomes in health and disease. Additionally, we identified 3 siblings carrying a homozygous missense variant of SPNS1. These patients exhibit clinical symptoms including developmental delay, neurological symptoms, intellectual disability, and cerebellar hypotrophy. Here, we show that LPC transport function of this variant was affected. Our study provides evidence for a critical role of SPNS1 for normal physiology.

## Results

### SPNS1 is expressed in late endosomes and lysosomes and is required for early development in mice.

**Figure 1:**
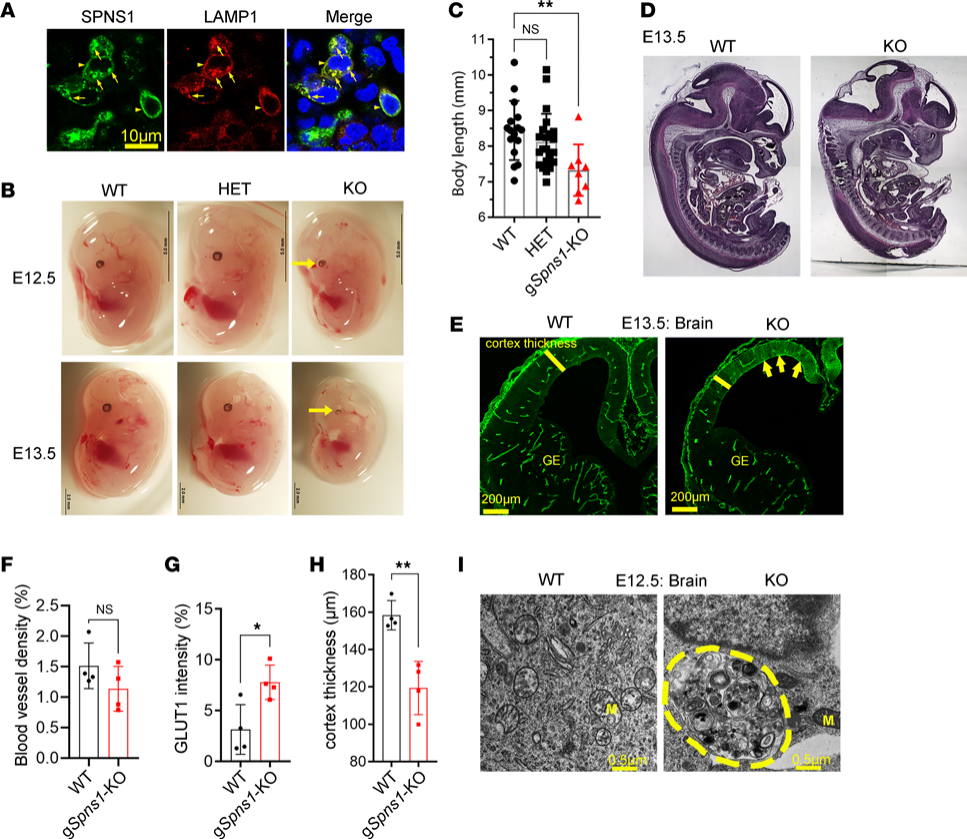
SPNS1 is a putative lysosomal transporter that is required for survival in mice. (**A**) Localization of SPNS1 in HEK293 cells. Human SPNS1 cDNA was cotransfected with LAMP1-RFP. Spns1 was colocalized with lysosomal marker LAMP1. (**B**) Representative images of E12.5 and E13.5 of WT, heterozygous (HET), and global Spns1 KO (KO) embryos. Arrows show maldevelopment of the eyes. (**C**) g*Spns1*-KO embryos were smaller than WT and HET controls. Each symbol represents 1 embryo (1-way ANOVA; *n* = 16 for WT, *n* = 22 for HET, *n* = 8 for KO; **P < 0.01; data are expressed as mean ± SD). (**D**) Gross anatomy of a control and g*Spns1*-KO embryos at E13.5. (**E**) Representative immunostaining with GLUT1 of the brain vasculature of controls and g*Spns1*-KO embryos. (**F**) Quantification of blood vessel density in the brain of control and g*Spns1*-KO embryos at E13.5. Each symbol represents 1 embryo (2-tailed unpaired *t* test; *n* = 4; *P < 0.05; data are expressed as mean ± SD). (**G**) Quantification of expression of GLUT1 in neocortical regions of g*Spns1*-KO embryos was significantly reduced. Each symbol represents 1 embryo (2-tailed unpaired *t* test; *n* = 4; **P < 0.01; data are expressed as mean ± SD). (**H**) Cortical thickness of g*Spns1*-KO embryos was reduced. Each symbol represents 1 embryo. *n* = 4 for each genotype. (**I**) Transmission electron microscopic images of brain sections of E12.5 WT and g*Spns1*-KO embryos. Accumulation of membranous structures (demarcated area) in the cytoplasm of brain cells of g*Spns1*-KO embryos. M, mitochondria. *n* = 3 per genotype.

SPNS1 shares a high sequence identity with SPNS2, an S1P transporter from endothelial cells (15). Previously, SPNS1 protein was also detected in the lysosomal protein fractions (14). To visualize the localization of SPNS1, we developed polyclonal antibodies to detect the expression and localization of SPNS1 in cells. We validated our antibodies using Western blotting (Supplemental Figure 1A; supplemental material available online with this article; https://doi.org/10.1172/jci.insight.175462DS1). In cell lines such as CHO and HEK293 cells, we detected the expression of SPNS1 with a single protein band of approximately 40 kDa (Supplemental Figure 1A). By performing immunostaining, we found that SPNS1 was colocalized with LAMP1, a lysosomal marker (Figure 1A, arrows). We also detected the expression of SPNS1 in or near the plasma membrane (Figure 1A, arrowheads). These data suggest that SPNS1 may first be translocated to the plasma membrane before being destined for the lysosomes. Furthermore, we tested whether SPNS1 was expressed in the endocytic vesicles. In HEK293 cells, when SPNS1 was coexpressed with Rab5, an early endosomal marker, we found that SPNS1 and Rab5 did not colocalize (Supplemental Figure 1B). Interestingly, SPNS1 was colocalized with Rab7, a marker for late endosomes (Supplemental Figure 1B). These results verify that SPNS1 was expressed in late endosomes and lysosomes in the cell lines.

The modeled structure of SPNS1 from AlphaFold2 shows that it resembles solute transporters and shares the high similarity with SPNS2 structure (Supplemental Figure 1C). SPNS1 function has not been studied in mouse models. To this end, we deleted *Spns1* in mice and found that global KO of *Spns1* (g*Spns1^–/–^,* hereafter g*Spns1*-KO) resulted in embryonic lethality between E12.5 and E13.5 with severe defects in the brain and eye (Figure 1B and Supplemental Figure 1D). We confirmed the deletion of *Spns1* in mice by genotyping and Western blot results (Supplemental Figure 1, E and F). Viable g*Spns1*-KO embryos were significantly smaller as compared with controls (Figure 1, B and C, and Supplemental Figure 1D). Nevertheless, the anatomy of the gS*pns1*-KO embryos was comparable with that of control embryos (Figure 1D). In the brain, the vascularization of the g*Spns1*-KO embryos appeared reduced with an increased expression of GLUT1 in the neocortices (Figure 1, E–G). Additionally, the cortical thickness was significantly reduced in the g*Spns1*-KO embryos (Figure 1H). By performing the transmission electron microscopic analysis of the g*Spns1*-KO brain, we found that there was an accumulation of membranous structures in the neural cells (Figure 1I and Supplemental Figure 1G). These results indicate that SPNS1 is required for embryonic development where its deletion results in several phenotypes in the brain, including the accumulation of membranous structures.

### Lack of SPNS1 results in accumulation of lysolipids.

**Figure 2:**
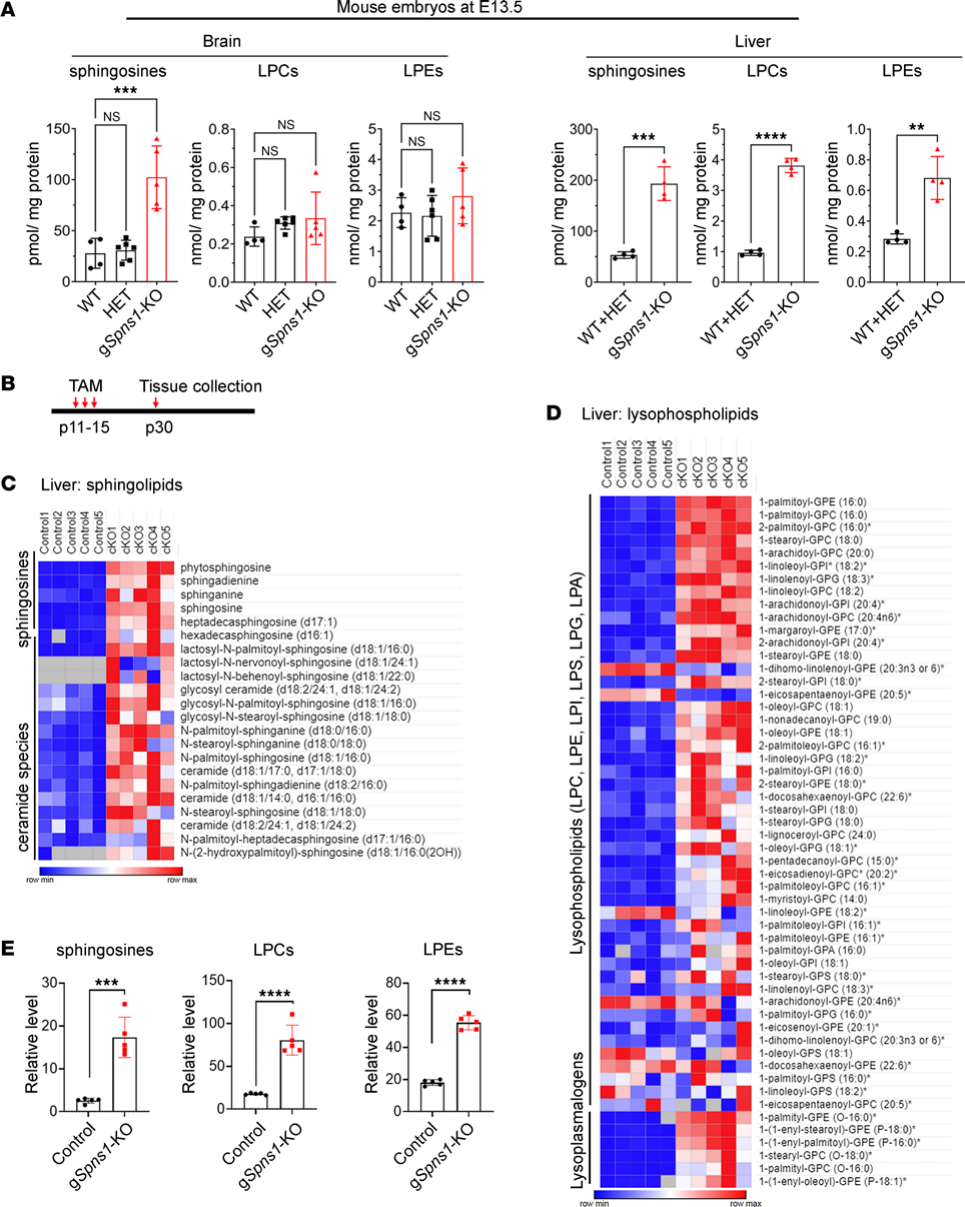
Accumulation of sphingosines and lysoglycerophospholipids in Spns1-KO mice. (**A**) Total levels of sphingosines, lysophosphatidylcholines (LPC), and lysophosphatidylethanolamines (LPE) from whole brains and livers of E13.5 WT, heterozygous (HET), and g*Spns1*-KO embryos. Note that the levels of sphingosine species were elevated in the brains and livers of Spns1-KO embryos, whereas the levels of LPC and LPE were increased in the fetal livers of the KO embryos. (1-way ANOVA for brain samples; *n* = 4 for WT, *n* = 6 for HET, and *n* = 4 for KO; 2-tailed unpaired *t* test for liver samples; *n* = 4 for each group; **P < 0.01, ***P < 0.001, ****P < 0.0001; data are expressed as mean ± SD). (**B**) Illustration of the postnatal deletion strategy of Spns1 using Rosa26Cre-ER^T2^ mice. (**C**–**E**) Metabolomic analysis of livers of control and g*Spns1*-cKO mice. *n* = 5 mice per genotype. (**C** and **D**) Heatmap of sphingolipid and lysoglycerophospholipid species. (**E**) Metabolomic analysis of sphingosines, LPCs, and LPEs of control and g*Spns1*-cKO mice (2-tailed unpaired *t* test; *n* = 5; ***P < 0.001, ****P < 0.0001; data are expressed as mean ± SD).

To reveal the identities of those membranous substances, we performed lipidomic analysis of brains and livers from g*Spns1*-KO and control embryos. We found that sphingosine species were significantly elevated in both brains and livers of the g*Spns1*-KO embryos, while the levels of ceramides, sphingomyelins, and phospholipids were unaffected (Figure 2A; Supplemental Figure 2, A and B; and Supplemental Data files 1 and 2). There was no significant increase in the levels of lysoglycerophospholipids such as LPC and LPE in the brains, but these lysolipids were dramatically increased in the fetal livers of g*Spns1*-KO embryos (Figure 2A and Supplemental Figure 2, A and B).

We noted that the levels of sphingosine accumulated in *Spns1*-KO embryos were comparable with the previous studies on NPC1 KOs (9). However, while the global KO of *NPC1* is still viable, the global KO of *Spns1* is embryonically lethal. Thus, accumulation of either sphingosine or lysoglycerophospholipids in g*Spns1*-KO embryos is unlikely to be the sole cause of the early lethality of *Spns1*-KO embryos.

To comprehensively assess the cellular substances accumulated due to the lack of SPNS1, we employed metabolomics to detect metabolites from major metabolic pathways. To achieve this goal, we generated conditional KO mice of *Spns1* (hereafter g*Spns1*-cKO mice) by crossing *Spns1^fl/fl^* and *Spns1^fl/fl^* with Rosa26Cre-ER^T2^ (Figure 2B and Supplemental Figure 3A). The *Spns1^fl/fl^* mice have a loxP site inserted on both sides of exon 3. Based on the induction of Cre by tamoxifen, the exon 3 is deleted, leading to a frameshift. Here, we deleted *Spns1* 2 weeks after birth and collected livers for metabolomics. The metabolomic analysis encompassed over 900 metabolites of all major metabolic pathways, such as amino acids, lipids, nucleotides, carbohydrates, cofactors, and vitamins (Supplemental Data files 3–8). This analysis identified many sphingolipid and lysoglycerophospholipid species that were significantly accumulated in the livers of g*Spns1*-cKO (*Spns1^fl/fl^ Cre*) mice compared with those of controls (*Spns1^fl/fl^* without *Cre*) (Figure 2, C and D). The accumulation of the lysolipids was specific since the levels of metabolites from the metabolic pathways for amino acids, nucleotides, and carbohydrates, as well as the levels of cofactors and vitamins, were largely unchanged in the livers of g*Spns1*-cKO mice (Supplemental Figure 3, B and C, and Supplemental Data files 3–8). Among elevated lysoglycerophospholipids, the levels of LPC, LPE, LPI, LPG, and lysoplasmalogens (including LPC-P and LPE-P) were significantly increased in the livers of g*Spns1*-cKO mice (Figure 2, C–E). In contrast, major glycerophospholipids such as PC, PE, phosphatidylserine (PS), phosphatidic acid (PA), phosphatidylinositol (PI), and plasmalogens in the livers of g*Spns1*-cKO mice remained comparable with those of the controls (Supplemental Data files 3–8). Consistent with the increased levels of sphingosines in embryonic tissues (Figure 2A), metabolomic analysis also enabled us to validate the increased levels of major sphingosine species (such as d18:1, d18:0, d16:1 sphingosine species) in g*Spns1*-cKO mice (Figure 2, C–E). Ceramides and lactosylceramides, but not sphingomyelins, were also significantly accumulated in the livers of g*Spns1*-cKO mice (Figure 2C, Supplemental Figure 3D, and Supplemental Data files 3–8). On the other hand, the levels of cholesterol and its related metabolites in the livers of the g*Spns1*-cKO mice were not elevated (Supplemental Figure 3E and Supplemental Data files 3–8). Consistently, the expression levels of lysosomal proteins important for cholesterol transport — including NPC1, NPC2, and LIMP2 in the livers of g*Spns1*-cKO mice — were unaltered, with the exception of the expression pattern of NPC2 (Supplemental Figure 3F). Therefore, these data verify that the postnatal deletion of *Spns1* in mice results in the accumulation of sphingolipids and lysoglycerophospholipids.

### SPNS1 is required for clearance of sphingosine and LPC by a transport mechanism.

**Figure 3:**
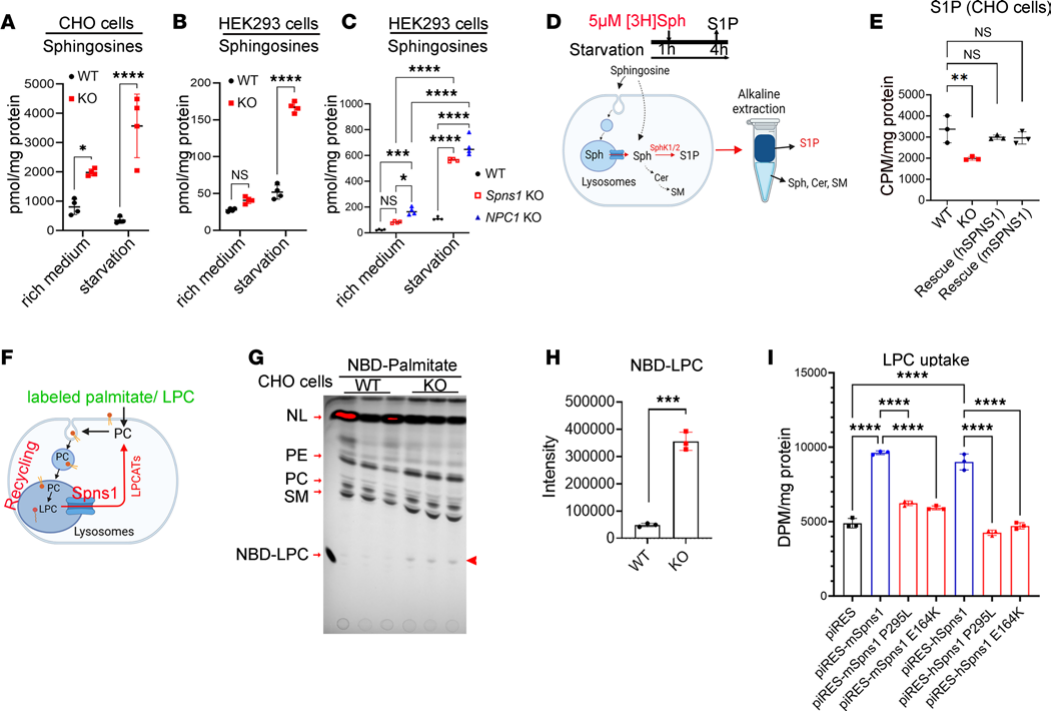
SPNS1 is required for sphingosine and LPC release from lysosomes. (**A**–**C**) Total levels of sphingosines from WT and Spns1-KO cells with or without starvation condition. WT and Spns1-KO CHO cells (**A**). WT and Spns1-KO HEK293 cells (**B**). WT, Spns1-KO, and NPC1-KO HEK293 cells (**C**). Each symbol represents 1 replicate (**A** and **B**, 2-tailed unpaired *t* test, *n* = 4; **C**, 1-way ANOVA, *n* = 4; *P < 0.05, ***P < 0.001, ****P < 0.0001; data are expressed as mean ± SD). (**D**) Illustration of [3-^3^H]-sphingosine transport assays. Cells were starved in medium without amino acids and serum for 1 hour, andthey were then added with radioactive sphingosine and incubated until collecting for radioactive S1P isolation from other sphingolipids (Sph, Cer, and SM) for quantification. S1P, Sphingosine-1-phosphate; Sph, Sphingosine; Cer, Ceramide; SM, Sphingomyelin. (**E**) Radioactive S1P levels (upper phase) from WT, Spns1-KO, and rescue CHO cells with mouse SPNS1 (mSPNS1) or human SPNS1 (hSPNS1). Each symbol represents 1 replicate. One-way ANOVA; *n* = 3; *P < 0.05, **P < 0.01, ***P < 0.001; data are expressed as mean ± SD). (**F**) Illustration of NBD-palmitate labeling experiment. (**G**) Thin-layer chromatography (TLC) analysis of NBD-labeled phospholipids after 24 hours of pulse-labeling with NBD-palmitate in WT and Spns1-KO CHO cells. Arrowhead indicates NBD-LPC band. NL, neutral lipids. (**H**) Quantification of NBD-LPC from the TLC plate (2-tailed unpaired *t* test; *n* = 3; ***P < 0.001; data are expressed as mean ± SD). (**I**) LPC transport activity of mouse (mSPNS1), human SPNS1 (hSPNS1) in HEK293 cells, and the missense mutation P295L. E164K was used as a control. piRESv2-EGFP was used as a mock control. Transfected HEK293 cells were incubated with 10 μM [^14^C] LPC for 20 minutes and collected for quantification of radioactive signals. Each symbol represents 1 replicate (1-way ANOVA; *n* = 3; ****P < 0.001; data are expressed as mean ± SD).

To gain further insights into the molecular functions of SPNS1, we generated *Spns1* KOs in HEK293 and CHO cells (*Spns1*-KO cells) (Supplemental Figure 4, A–C) and employed the cells for biochemical transport assays. We validated that sphingosine species were also accumulated in these *Spns1*-KO cells, especially under starvation conditions (Figure 3, A and B, and Supplemental Data files 9 and 10). Accumulation of sphingosines has been reported in NPC1-deficient cells. Thus, we included *NPC1-*KO HEK293 cells (*NPC1*-KO cells) in our lipidomic analysis for comparison. Interestingly, we found that the deletion of *Spns1* caused an increased level of sphingosines similar to that found in *NPC1*-KO cells (Figure 3C and Supplemental Data file 11). Nevertheless, NPC1/NPC2 expression in *Spns1*-KO cells was unchanged, as was expression of SPNS1 in *NPC1*-KO cells (Supplemental Figure 4A). Therefore, it is likely that SPNS1 regulates sphingosine transport in the lysosomes independently of NPC1.

Next, we tested whether SPNS1 mediates sphingosine uptake using a cell surface assay. However, there was no significant difference for sphingosine uptake between control and KO cells (Supplemental Figure 4, D and E). This was probably due to the properties of sphingosine that enable it to be quickly taken up by the cells via endocytosis and diffusion, thus making it challenging to test for the direct uptake of sphingosine (16–20). Therefore, we established an indirect transport assay where we measured sphingosine release from the lysosomes via measurement of S1P synthesis in the cytoplasm (Figure 3D). A part of exogenous sphingosine is delivered to the lysosomes via endocytosis. Indeed, incubating WT CHO cells with 1% Methyl-β-cyclodextrin (MCD) to inhibit endocytosis (21) significantly reduced [3-^3^H]-sphingosine uptake (Supplemental Figure 4F). By employing this assay, we showed that lysosomal sphingosine release for S1P synthesis was significantly reduced in both CHO and HEK293 cells with the KO of SPNS1 (Figure 3E and Supplemental Figure 4, G–I). Although these results do not show the direct transport of sphingosine by SPNS1, they strongly suggest that the transporter is required for sphingosine release from the lysosomes.

The accumulation of lysoglycerophospholipids in our results suggests that SPNS1 also transports these lysolipids. To gain further insights into the potential role of SPNS1 as a transporter for the lysoglycerophospholipids, we incubated WT and *Spns1*-KO CHO cells with NBD-palmitate and subsequently analyzed NBD-labeled phospholipids by thin-layer chromatography (TLC) (Figure 3F). We found that, in *Spns1*-KO cells, NBD-LPC accumulated more than in control cells (Figure 3, G and H). While we were testing the LPC transport activity on SPNS1, 2 new studies coincidentally reported that SPNS1 is a transporter for LPC (22, 23). Adapting the cell-surface uptake assay established by He et al., we were also able to show direct LPC uptake by both human SPNS1 and mouse SPNS1 when they were overexpressed in HEK293 cells (Figure 3I). These results confirm that SPNS1 is a transporter for LPC from the lysosomes.

Interestingly, we identified a missense variant in the coding sequence of SPNS1 (Chr.16: 28993296, c.C884T:p.P295L), which changed proline at the position of 295 to leucine (P295L) in 3 patients of the same consanguineous family who have severe neurodevelopmental symptoms (Supplemental Figure 5, A and B; Supplemental Methods; and Supplemental Table 1). The family also has 1 unaffected child who was not homozygous for this variant. Linkage analysis identified only 2 chromosomal regions, in which a total of 5 different potentially homozygous variants were identified (Supplemental Table 1). The variant in *SPNS1* was by far the most likely candidate, as 3 of the 5 mutations have been reported in homozygous form in gnomAD (Supplemental Table 1), which removes pediatric genetic disease cases, and the only other remaining mutation is not considered likely to be pathological by 3 bioinformatic programs: SIFT, CADD, and REVEL (Supplemental Table 1).

Since the proline 295 residue is highly conserved between mouse and human SPNS1 (Supplemental Figure 5, C and D), we tested LPC transport activity of both human and mouse P295L mutants. We also included an E164K mutant because this conserved glutamate residue has been shown to be important for brain development in *Drosophila* (24). Using the cellular transport assay, we found that both the P295L and the E164K mutation exhibited significantly reduced LPC transport activity (Figure 3I and Supplemental Figure 5, E and F). Collectively, these results show that SPNS1 is required for the clearance of the lysolipids from lysosomes by a transport mechanism and that a defective release of these lysolipids from the lysosomes via SPNS1 might be linked to the symptoms in these patients.

### Deficiency of SPNS1 results in phenotypes reminiscent of lysosomal storage diseases in mice.

**Figure 4:**
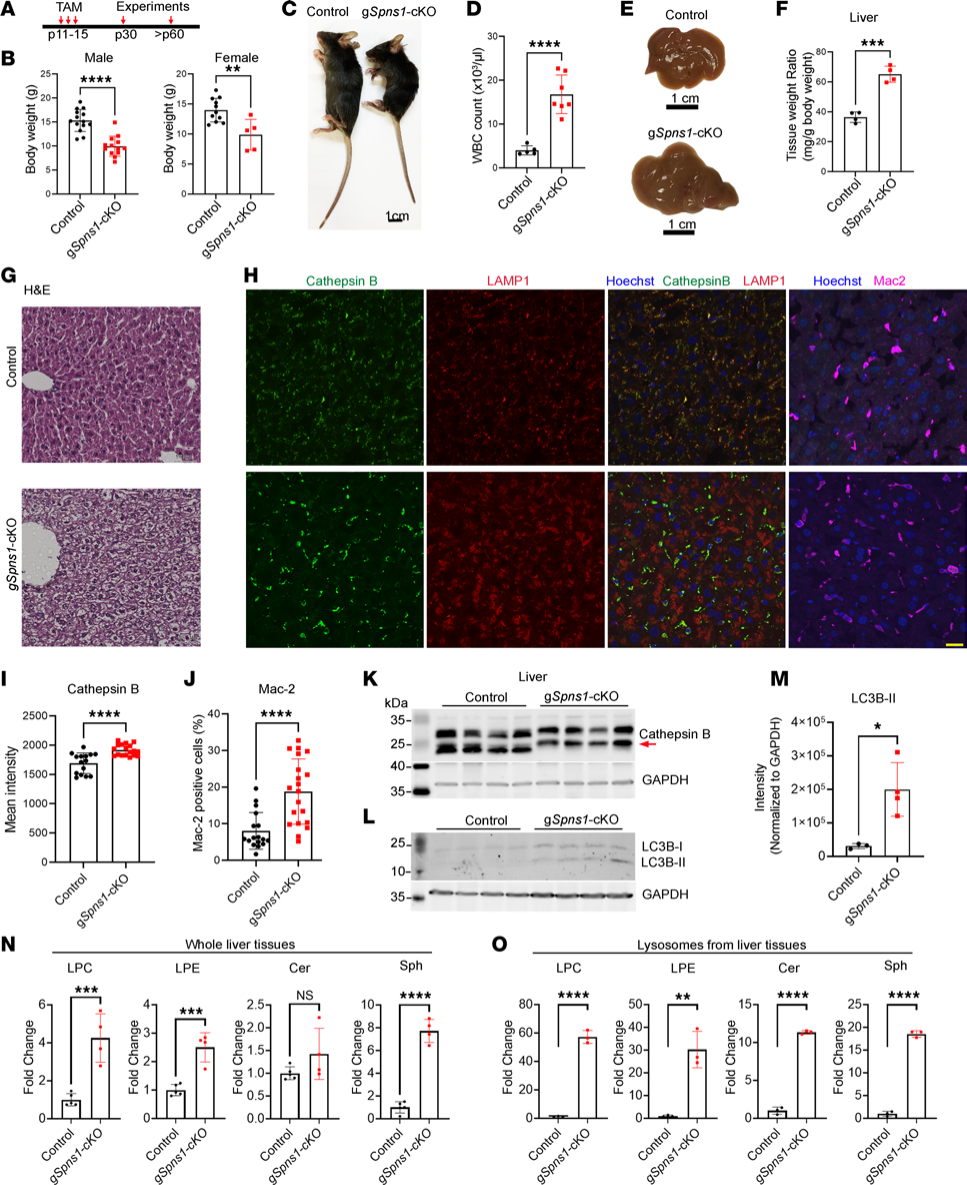
Spns1-KO mice exhibit lysosomal storage phenotypes. (**A**) Illustration of the postnatal deletion strategy of Spns1 using Rosa26Cre-ER^T2^ mice. (**B**) Reduction of body weights of g*Spns1*-cKO male and female mice 2 weeks after tamoxifen treatment. Each symbol represents 1 mouse. (**C**) Representative images of a control and a g*Spns1*-cKO mouse. (**D**) Increased number of white blood cells (WBC) in g*Spns1*-cKO mice compared with controls. Each symbol represents 1 mouse. (**E**) Representative images of livers from a control and a g*Spns1*-cKO mouse. (**F**) Increased liver weights in g*Spns1*-cKO mice compared with the controls of the same age. Each symbol represents 1 mouse. (**G**) Representative images of H&E staining of liver sections from control and g*Spns1*-cKO mice. Livers of g*Spns1*-cKO mice were presented a foamy phenotype. *n* = 3 per genotype. Scale bar: 50 μm. (**H**) Representative of immunostaining images of liver sections from control and g*Spns1*-cKO mice with cathepsin B and LAMP1 or with Mac-2 for macrophages. Scale bar: 20 μm. *n* = 3 per genotype. (**I**) Quantification of cathepsin B fluorescence intensity from **H**. (**J**) Numeration of Mac-2^+^ cells from **H**. Each symbol represents one section from *n* = 3 mice per genotype. (**K** and **L**) Western blot analysis of cathepsin B and LC3B proteins from whole liver protein lysates of control and g*Spns1*-cKO mice. Cathepsin B processing was defective in the livers of g*Spns1*-cKO mice (arrow). *n* = 4 per genotype. (**M**) Quantification of total LC3B-II bands from **L**. (**N** and **O**) The levels of lysophosphatidylcholine (LPC), lysophosphatidylethanolamine (LPE), ceramides, and sphingosines from whole livers and lysosomal fractions (F1 and F2) of control and g*Spns1*-cKO mice, respectively. *n* = 4–5 for whole liver. *n* = 3 per genotype for lysosomes. *P < 0.05; **P < 0.01; ***P < 0.001; ****P < 0.0001. Data are expressed as mean ± SD. Statistical significance was determined by 2-tailed unpaired *t* test.

Global KO of *Spns1* results in early embryonic lethality, indicating that SPNS1 plays a critical role in the early developmental stages. To gain further insights into the physiological roles of SPNS1 in adulthood, we characterized the cKO mice of *Spns1* after birth (Figure 4, A–F). Postnatal deletion of *Spns1* resulted in a significant reduction of body weight in both male and female mice 2 weeks after injection of tamoxifen (Figure 4, B and C). The increase in white blood cell (WBC) counts was reported in models of lysosomal storage diseases (19, 25). Similarly, we found that the WBC count of g*Spns1*-cKO was significantly elevated, suggestive of increased inflammation in these mice after a 2-month deletion (Figure 4D). Interestingly, we observed that livers of g*Spns1*-cKO mice exhibited hyperplasia (Figure 4, E and F). Histological assessment of the liver sections revealed that hepatocytes exhibited a foamy phenotype that is often observed in lysosomal storage diseases (Figure 4G). These observations were not reported in the knockdown of SPNS1 in the liver, perhaps due to the partial deletion of the gene by AAV approach (22). The expression of cathepsin B was clustered around the nucleus with increased fluorescence intensity in hepatic cells of g*Spns1*-cKO mice (Figure 4, H and I, and Supplemental Figure 6A). Furthermore, there were increased numbers of macrophages in the livers of g*Spns1*-cKO mice as compared with controls (Figure 4, H and J). Interestingly, we found that the maturation and deglycosylation of the cathepsin B was defective (Figure 4K), suggestive of lysosomal defects (26). In addition, the expression of LC3B-II was slightly but significantly increased in the livers of g*Spns1*-cKO mice compared with the controls (Figure 4, L and M). These results point to a possibility that the pathological conditions in the livers of g*Spns1*-cKO mice could be linked to the defects in lysolipid clearance from the lysosomes. Thus, we enriched lysosomes from the livers of g*Spns1*-cKO and control mice and performed lipidomic analysis for sphingolipids and lysoglycerophospholipids (Supplemental Figure 6, B and C). We found that these lysolipids accumulated to significantly high levels in the lysosomal fractions from livers of g*Spns1*-cKO mice (Figure 4, N and O; Supplemental Figure 6D; and Supplemental Data files 12–15). These results reaffirm that SPNS1 is indispensable for the transport of these lysolipids out of the lysosomes. Collectively, our findings indicate that the deficiency of SPNS1 results in lysolipid accumulation in the lysosomes, causing the pathological conditions that resemble lysosomal storage diseases.

### Lipid accumulation due to Spns1 deletion leads to multiple changes, including the PI3K/AKT signaling pathway in the liver.

**Figure 5:**
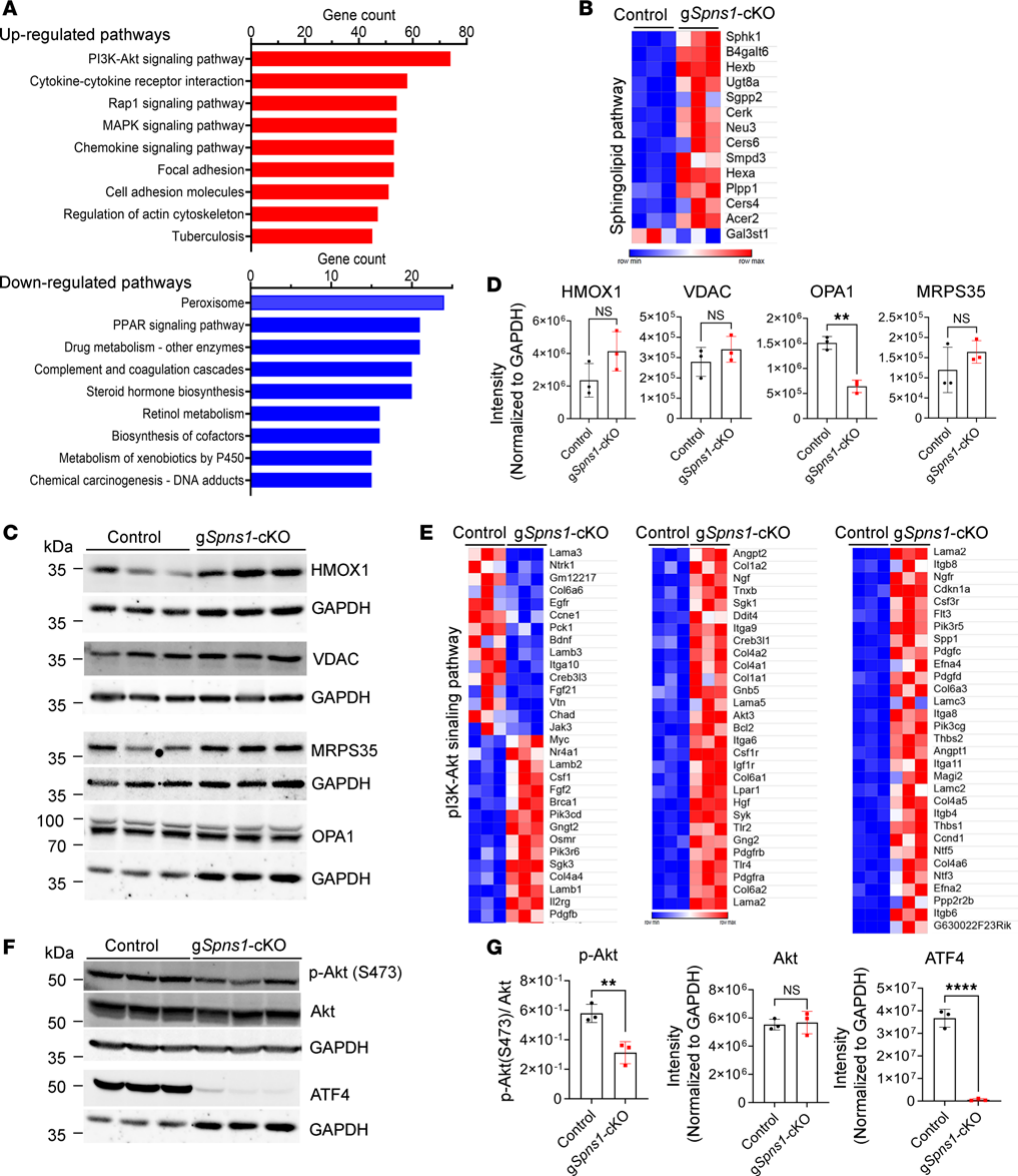
Alteration of ATF4 and p-Akt signaling in livers of Spns1-KO mice. (**A**) RNA-Seq analysis of control and g*Spns1*-cKO livers. The graphs illustrate the most significantly affected pathways in g*Spns1*-KO livers identified by the Kyoto Encyclopedia of Genes and Genomes (KEGG) pathway analysis. (**B**) Increased expression levels of mRNA for genes in sphingolipid metabolism. Expression of the genes in the sphingolipid pathway was significantly upregulated in livers of g*Spns1*-cKO mice. (**C**) Western blot analysis of mitochondrial markers: HMOX1, VDAC, MRPS35, and OPA1 in livers of control and g*Spns1*-cKO. *n* = 3 per genotype. (**D**) Quantification of expression levels of protein bands in **C**. Expression of OPA1 was significantly reduced in g*Spns1*-cKO livers. (**E**) Differential expression levels of genes in the PI3K/AKT signaling pathway. Many genes in the PI3K/AKT signaling pathway were differentially expressed in the g*Spns1*-cKO livers. (**F** and **G**) Western blot analysis of ATF4, p-Akt, and total Akt in control and g*Spns1*-CKO livers. *n* = 3 per genotype. OPA1 and ATF4 were probed on a same membrane and shared GAPDH control. (**G**) Quantification of total expressions of proteins in **F** after normalization to GAPDH. **P < 0.01; ****P < 0.0001. Data are expressed as mean ± SD. Statistical significance was determined by 2-tailed unpaired *t* test.

To gain insights into the pathways that are altered by lipid accumulation, we performed RNA-Seq of the livers of *Spns1*-KO mice. Our results show that there were multiple changes in gene expression from several biological pathways (Figure 5A and Supplemental Data file 16). Interestingly, the transcriptional levels of several genes in the sphingolipid metabolism pathway were increased (Figure 5B). The increased expression of the genes in the sphingolipid pathway might reflect a deficient level of sphingosine in the cytoplasm. Notably, there was an increased expression of inflammatory and cell adhesion genes in line with the increased infiltration of immune cells to the livers of *Spns1*-KO mice (Figure 5A). Hepatic lysosomal lipid accumulation appeared particularly detrimental to the expression of peroxisomal genes, which include the genes important for β-oxidation (Figure 5A). However, the expression levels of mitochondrial proteins such as VDAC, heme oxygenase-1 (HMOX1), and MRPS35 were unaltered, with the exception of the expression reduction of OPA1 (Figure 5, C and D). Therefore, it is unlikely that lipid accumulation exerts a strong effect to mitochondrial functions in the livers of *Spns1*-KO mice. Among the top upregulated pathways in the livers of *Spns1*-KO mice, many genes in the PI3K/AKT signaling pathway were differentially affected (Figure 5E). Interestingly, the expression level of phosphorylated AKT (p-AKT), a key regulator in the pathway, was significantly reduced in the livers of *Spns1*-KO mice (Figure 5, F and G). Furthermore, we found that the expression level of ATF4 was also dramatically reduced in the livers of *Spns1*-KO mice compared with that of controls (Figure 5, F and G). In addition to its role in cellular stress response, ATF4 is also induced by metabolic changes such as feeding, which involves the insulin signaling via PI3K/AKT pathway (27, 28). These changes are likely due to SPNS1 inhibition in hepatocytes because expression levels of p-AKT and ATF4 were also decreased in *Spns1*-KO cells but were not in *NPC1*-KO cells (Supplemental Figure 7, A and B). Together, these results reveal the detrimental effects of lysolipid accumulation in the lysosomes due to *Spns1* deletion that negatively affects multiple biological pathways, including the PI3K/AKT/ATF4 signaling pathway in the liver.

## Discussion

SPNS1 was first discovered in flies as a gene involved in mating behaviors (24). The mutant flies of the same gene (namely *Spin*) exhibited aberrant apoptosis of neurons and glial cells (24). SPIN-mutant flies showed an accumulation of autofluorescent, lipofuscin-like pigments in the brain at the early stage of development (24, 25, 29, 30), shown by lipidomic analysis to be ceramides and sphingosines (29). In zebrafish, the deletion of *Spns1* resulted in lethality. SPNS1-mutant fish exhibited accumulation of lipofuscin-like structures in the muscle and liver and had accelerated signs of aging with shorter life spans (30). The mutant fish also exhibited delayed clearance of autophagosomal contents (31). Consistent with the essential role of SPNS1 for survival from flies to fish, we showed that global KO of *Spns1* in mice resulted in embryonic lethality between E12.5 and E13.5, with abnormal vesicle structures in the KO embryonic brains.

The nature of the lipofuscin-related phenotypes in animals with SPNS1 deficiency was uncharacterized. Several other studies have suggested that SPNS1 is involved in transporting sugars and amino acids (32, 33). However, these studies fail to explain the nature of lipofuscin structures and lipid accumulation in SPNS1-deficient cells and animals (29). Consistent with findings in mutant flies (29), we found that KO of *Spns1* in mice resulted in the accumulation of sphingolipids, especially sphingosines. Sphingosine is protonated in the lysosomal lumen’s acidic compartment, preventing it from diffusing via the membranes. Our biochemical results strongly suggest that SPNS1 is needed for the release of lysosomal sphingosines. Recently, SPNS1 was reported as a transporter for LPC and LPE from the lysosomes (22, 23). Consistent with these recent findings, we also showed that the lack of SPNS1 resulted in remarkably elevated levels of lysoglycerophospholipids such as LPC, LPE, LPG, and lysoplasmalogens. Our biochemical experiments also corroborated that the expression of SPNS1 is necessary for LPC transport using cell-surface transport assay (22). Interestingly, both our data and the data from He et al. show that the lack of SPNS1 caused elevated levels of sphingosines and ceramides in mice (22), although this was not observed by Scharenberg et al. (23). Furthermore, He et al. employed the cell-surface assay and were unable to show that SPNS1 transports sphingosine (22). It should be noted that exogenous sphingosine is rapidly taken up by cells (16–20). Thus, it would be difficult to tease out the import effect mediated by SPNS1. In contrast, using an indirect transport assay, we obtained strong evidence to show that SPNS1 is required for sphingosine transport from the lysosomes. Nevertheless, a direct sphingosine transport by SPNS1 remains to be established.

At this point, it is evident that the lack of SPNS1 results in the accumulation of both lysoglycerophospholipids and sphingolipids in the lysosomes. However, the major lipid that leads to the early lethality and phenotypic changes observed in the KO mice of *Spns1* remains elusive. Complex glycerophospholipids such as PC and PE are hydrolyzed by lysosomal phospholipase A2, mainly PLA2G15 (also known as LPLA2), to liberate their respective lysoglycerophospholipids (34). However, the lack of lysosomal hydrolysis of these glycerophospholipids appears less detrimental to the mice, because mice lacking PLA2G15 that results in the accumulation of phospholipids, but not sphingolipids in the lysosomes, are viable and only exhibit the phenotypes at later ages (34, 35). It is also worth noting that lysoglycerophospholipids can be further hydrolyzed into free fatty acids and glycerophosphodiesters such as GPC and GPE in the lysosomes. Indeed, a recent study showed that the protein encoded by *CLN3*, a gene mutated in Batten disease, might be responsible for transport of these glycerophosphodiesters from lysosomes (7). Our metabolomic results show that the lack of SPNS1 does not cause the accumulation of these glycerophosphodiesters, implying that the release of these by-products from the hydrolysis of lysoglycerophospholipids is still active in *Spns1*-KO cells. Thus, it seems that the accumulation of lysoglycerophospholipids in the lysosomes causes milder pathological conditions compared with that of sphingolipids. Interestingly, we observed that sphingosines, but not LPC and LPE, were accumulated in the brain of *Spns1*-KO embryos. Thus, it might be that the accumulation of lysoglycerophospholipids and/or sphingolipids due to *Spns1* deletion is cell type dependent. The accumulation of sphingolipids has been shown to be detrimental to the cells and animal models, notably in models of SLSD (2, 10, 36). In line with the essential role of sphingosine export from the lysosome, KO of acid ceramidase (*Asah1*), the lysosomal ceramidase in mice, causes embryonic lethality (37). Deficiency of ASAH1 in adult mice also results in severe phenotypes reminiscent of phenotypes of *Spns1*–cKO mice (25). These findings imply that the release of sphingosines from the lysosomes likely mediated by SPNS1 is a critical step to avoid lysosomal defects. Nevertheless, a remarkable amount of lysoglycerophospholipids, including LPC and lysoplasmalogens accumulated in the lysosomes of SPNS1 KOs, is also predicted to exert additional detrimental effects. It would be interesting for future studies to reveal the primary lysolipids that drive the accumulation of other lysolipids in the lysosomes of *Spns1*-deficient cells.

Accumulation of sphingosines causes various defects in the cells such as dysregulation of calcium homeostasis or alteration of insulin signaling via inhibition of phosphorylation of Akt (38–40). Consistently, we detect a defect in the PI3K/AKT signaling pathway in the liver of *Spns1*-KO mice. A significantly decreased level of ATF4, which is a downstream player in PI3K/AKT signaling pathway, might be a part in the pathogenesis of the liver. ATF4 is important for adaptation to the metabolic stress and feeding conditions (41). Inhibition of ATF4 expression is expected to reduce oxidative stress and inflammation and cause liver injury in mice (42, 43). Therefore, accumulation of sphingosines in the livers of *Spns1*-KO mice might be a major part of the pathogenesis, suggesting that lowering sphingosine accumulation would have beneficial effects on the functions of liver in lysosomal storage diseases.

We identified 3 patients with a homozygous variant of SPNS1 who exhibit clinical symptoms, including neurodevelopmental delay, intellectual disability, and speech problems, but lack the vision problems. This change significantly affects LPC transport function of SPNS1 in vitro, likely associating a defect of SPNS1 function to the phenotypes in the patients. However, as the 3 siblings also carry other variants, and no additional families with *SPNS1* variants have been identified to date, the genetic to phenotypic association needs to be confirmed. Since deficiency of *Spns1* in mice causes lysosomal storage disease, we speculate that a defect in the function of *SPNS1* in humans also results in lysosomal storage disease.

In summary, our findings show that SPNS1 plays a crucial role during embryonic development. Lack of SPNS1 results in pathological conditions similar to lysosomal storage diseases in mice. The severe phenotypes of *Spns1*-KO mice are likely due to a defective transport of lysolipids out of the lysosomes. Thus, the identification of SPNS1 as a transporter for lysolipids provides a foundation for future study on the transport mechanisms of these lysolipids from the lysosomes.

## Methods

### Sex as a biological variable.

In our study, both male and female animals exhibited similar findings.

### Mice.

All the mice were in C57BL/6 background. The heterozygous of *Spns1* was obtained from International Mouse Phenotyping Consortium (IMPC, allele Spns1^tm1e[EUCOMM]Wtsi^). To generate the g*Spns1*-KO, heterozygous (*Spns1^+/–^*) *Spns1* mice were intercrossed, and the embryos were collected from timed-mated pregnant females. On the day of the experiments, pregnant mice were anesthetized by CO_2_, and then the embryos were collected. The WT, heterozygous, and homozygous KO embryos of the same litter were collected for experiments. Tail tips were collected for genotyping. The genotyping primers are listed in the Supplemental Methods.

The cKO mice were generated by the Transgenic and Gene Targeting Facility (TGTF), at the Cancer Science Institute of Singapore (CSI). First, a loxP site was inserted on both sides of the exon 3 using CRISPR/Cas9 technology. To generate the postnatal deletion of *Spns1* in the whole body (hereafter g*Spns1*-cKO), conditional *Spns1^fl/fl^* mice were bred with Rosa26Cre-ER^T2^ mice. The Cre expression was induced by i.p. injection of tamoxifen (TAM) at a dose of 50 mg/kg body weight at P11, P13, and P15. TAM was prepared by dissolving in corn oil with a 20 mg/mL concentration. *Spns1^fl/fl^* littermates were used as controls.

### Cell culture.

HEK293 and CHO cells were cultured in DMEM (Thermo Fisher Scientific, 11995065) media supplemented with 10% fetal bovine serum (Thermo Fisher Scientific, 10270106) and 1% penicillin/streptomycin (Thermo Fisher Scientific, 15140122) in the 37°C incubator with 5% CO_2_. The cells were starved in the DMEM without FBS and without amino acid (Wako, 048-33575).

### CRISPR/Cas9 gene deletion and rescue.

*Spns1* coding sequence in HEK293 and CHO cells was targeted by CRISPR/Cas9 technology. Briefly, individual gRNA generated using CRISPR/Cas9 v2 plasmid (Addgene, 52961) was transfected to HEK293 or CHO cells. The following gRNAs were used: 5′-ATTCCTGCTGCGTTCCCGCGTGG-3′ (for HEK293 cells), 5′-GCACGAGATCCCGGACCGCGAGG-3′ and 5′-TGATGCGCTGCAGACCCTCGCGG-3′ (for CHO cells). After 24 hours of transfection using Lipofectamine 2000 (Invitrogen), single cells were sorted by flow cytometry onto 96-well plates. After expansion, these *Spns1*-KO clones were subjected to Western blot analysis to detect SPNS1 protein using in-house–generated polyclonal antibodies. Clones with a complete absence of SPNS1 protein band were selected for experiments. To rescue SPNS1 in *Spns1*-KO HEK293 cells, mutant *Spns1* gene was knocked in *Spns1* locus using CRISPR/Cas9 technology. To rescue SPNS1 in KO CHO cells, the following mixture was used to transfect to the cells: 1 μg of FUGW plasmid, 1 μg of pMDLg/pRRE, 1 μg of pRSV-Rev, 0.5 μg of MD2G. All the plasmids were acquired from Addgene. After infection, Zeocin (Thermo Fisher Scientific) was added to the medium for the screening of the cell lines. Expression of SPNS1 was confirmed by Western blot in rescued cell lines.

### Lipidomic analysis.

Lipid extraction methods for tissues, cells, and lysosome fractions are described in the Supplemental Methods. Briefly, 200 μL butanol/methanol (1:1, v/v) containing internal standards was added in each sample to extract lipids, including phospholipids and sphingolipids. The extracted lipids were then separated using a reverse phase column (for sphingolipids) or a Hilic column (for phospholipids). Detailed protocols for lipid quantification and data processing are written in the Supplemental Methods.

### Enrichment of lysosomes from liver tissues.

Lysosomes from mouse livers were isolated using the tyloxapol method that has been previously described (44). The detailed protocol is provided in the Supplemental Methods. Briefly, control and g*Spns1*-cKO mice were injected with 4 μL/g body weight of a 17% (w/v in 0.9% NaCl) Triton WR1339 solution (Sigma-Aldrich) for 3 days prior to fractionation of lysosomes. The nonperfused livers were homogenized in 0.25 M sucrose and were then centrifuged at 1,000*g* for 10 minutes at 4°C to collect the postnuclear supernatant (PNS). Lysosomes were extracted from PNS by sucrose gradient using ultracentrifugation at 56,000*g* for 7 minutes (Hitachi CP90WX). The first 2 fractions containing lysosomes were collected for analysis by Western blot and lipidomics.

### Immunostaining and confocal microscopic analysis.

Embryos were collected at E12.5 and E13.5 from timed-mated heterozygous *Spns1* mice and then fixed in 4% PFA prepared in PBS at 4°C overnight. The next day, embryos were changed to 15% sucrose for 24 hours and then 30% sucrose for 24 hours prior to being embedded in OCT. Embryonic brains were dissected into 25–30 μm sections for staining by immunofluorescence (IF). Briefly, sections were washed twice with PBS for 30 minutes. Then, sections were permeabilized in 0.1% Triton X-100 in PBS for 2 hours, followed by the incubation with a blocking solution containing 1% BSA, 2.5% normal goat serum (NGS), and 0.1% Triton X-100 in PBS for 1 hour. Next, sections were incubated with anti–mouse GLUT1 (Abcam, ab40084, 1:200) at 4°C overnight, followed by 3 washes in PBS at 5-minute intervals. Then, the goat anti–mouse antibody (Alexa Fluor 488, Thermo Fisher Scientific, A11034, 1:500) was added to the slides for 1-hour incubation at room temperature to visualize the GLUT1 signal. The sections were then washed twice with PBS (5-minute intervals) and then stained with Hoechst 33342 (Thermo Fisher Scientific, 62249, 1:1,000 in PBS) for 15 minutes at room temperature. After washing the slides twice with PBS, the sections were mounted with mounting media. The sections were imaged using a Zeiss LSM710 confocal microscope. Similarly, immunostaining for liver sections from control and g*Spns1*-cKO mice was also performed (details are provided in Supplemental Methods). For visualization of lysosomes, cathepsin B (31718S, Cell Signaling Technology) and LAMP1 (Abcam, ab25245) antibodies were used. For visualization of macrophages, Mac-2 antibody (125401, BioLegend) was used. The IF quantification method is described in the Supplemental Methods.

### Sphingosine transport assay with and without inhibition of autophagy/endocytosis.

For sphingosine transport assays, WT HEK293, CHO, or corresponding *Spns1*-KO cells were used. Before transport assays, the confluent cells were starved in DMEM without amino acids and serum (Wako, 048-33575) for 1 hour. Next, the cells were incubated with 5 μM [3-^3^H]-Sph (American Radiochemicals) prepared in 12% BSA in the same starvation medium for 2 or 4 hours. The cells were washed once with DMEM medium containing 0.5% BSA and lysed in RIPA buffer for lipid extraction. [3-^3^H]-S1P was then isolated from the cell lysates using alkaline extraction methods (45, 46). This method recovers approximately 95% S1P in the upper phase. The radioactive signals in the upper phase (containing [3-^3^H]-S1P) and lower phase (containing [3-^3^H]-sphingosine, [3-^3^H]-ceramides, [3-^3^H]-SM) were quantified by a scintillation counter (PerkinElmer Tri-Carb Liquid Scintillation Analyzer).

For sphingosine transport assays with MCD inhibition, WT CHO cells were incubated in starvation medium containing 1% MCD (w/v), for 1 hour. The cells were then incubated with 5 μM [3-^3^H]-Sph prepared in 12% BSA in the same starvation medium containing 1% MCD for 30 minutes. The cells were washed once with plain DMEM containing 0.5% BSA and lysed in 500 μL RIPA buffer for scintillation quantification.

### NBD-palmitate labeling assay.

For NBD-palmitate labeling, 10 μM NBD-palmitate (Avanti, 810105P) solubilized in 12% BSA was added to the cells for 24 hours in the growth medium. Lipids from cells were extracted twice with 500 μL HIP buffer (Hexane, 2-propanol, 3:2, v/v) and dried under nitrogen gas. Phospholipids were separated in a TLC plate using chloroform/methanol/H_2_O (65:25:4, v/v). Fluorescent lipids on TLC plates were visualized by ChemiDoc (Bio-Rad) and quantified using ImageLab.

### Hematological analysis.

Blood samples were collected into EDTA-K2–coated tubes from control and g*Spns1*-cKO mice and were directly analyzed using Celltex-a MEK-6400 (Nihon Kohden).

### Metabolomics.

For metabolomics, we collected 50–100 mg tissues from PBS-perfused livers of *Spns1^fl/fl^*-Rosa26Cre-ER^T2^ (g*Spns1*-cKO) mice and control mice. The untargeted metabolomics was performed by Metabolon. Detail methods for metabolomic analysis from the company’s description are described in Supplemental Methods. Heatmaps of differential levels of metabolites were generated using Morpheus (https://software.broadinstitute.org/morpheus).

### Electron microscope.

WT (*n* = 3) and global KO embryos of *Spns1* (*n* = 3) at E12.5–E13.5 were collected and fixed in 2.5% glutaraldehyde for 24 hours at room temperature. Mouse embryo sections were prepared and were postfixed with 1% osmium tetroxide for 1 hour and dehydrated using a serial ethanol concentration. Images were taken using a transmission electron microscope.

### Patients.

Whole-exome sequence analysis is described in Supplemental Methods. The family discussed here is of consanguineous Turkish background. The parents are first cousins, with 3 affected children and 1 unaffected child. All 3 children were born by spontaneous uncomplicated vaginal delivery. They showed developmental delay (e.g., walking around age 2; 2 of the 3 children spoke only by age 4, while 1 child started speaking age 2). They learned to read and write between the ages of 12 and 16 and, at ages 12–20, display dysarthric speech, mild cerebellar signs, and mild intellectual impairment, with the youngest currently showing less impairment than the older children. More description of the patients is provided in Supplemental Methods. The human geneticist and mouse biologist found each other through GeneMatcher (47).

### Statistics.

Data were analyzed using GraphPad Prism 9.0 software. Statistical significance was calculated using the Student’s *t* test (2-tailed), Welch’s 2-tailed *t* test, and 1-way or 2-way ANOVA for multiple comparisons. *P* < 0.05 was considered as significant and indicated by asterisks.

### Study approval.

Patients gave written informed consent for participation in the University of Michigan’s program to identify novel genes in neurological disorders (IRB HUM00041414). All animal procedures were reviewed and approved by IACUC protocols (BR19-0633 and R019-567).

### Data availability.

All data are included in the article and Supplemental Information. Values for all data points in the graph are in the Supporting Data Values file. The RNA-Seq data were uploaded to Gene Expression Omnibus with the accession no. GSE240323.

## Author contributions

HTTH performed all in vivo experiments. XTAN, TQN, and DTN helped with some mouse work. HTTH created KO cell lines, and LKV made rescue in CHO cell lines. LKV and HTTH performed transport assays. HTTH, DTN, PYL, JO, ACG, and MRW performed lipidomics. SL, HTTH, and NCPL performed Western blot. HTTH performed H&E staining. HTTH, XTAN, and SL performed immunofluorescence staining and confocal imaging. HTTH and SB made mutants. ES, ZY, and MB provided clinical data. YJW and WYO performed electron microscopy experiment. LNN conceived, designed, and supervised the study and experiments. LNN and HTTH analyzed data, prepared the figures, and wrote the paper. LNN acquired funding for the project.

## Supplementary Material

Supplemental data

Supplemental tables 1-16

Supporting data values

## Version 1 03/07/2024

In-Press Preview

## Version 2 04/22/2024

Electronic publication
