# Supplementary material for: Lack of SPNS1 results in accumulation of lysolipids and lysosomal storage disease in mouse models
Source: JCI Insight. 2024 Mar 7;9(8):e175462. doi: 10.1172/jci.insight.175462 (PMC11141868; doi:10.1172/jci.insight.175462)
Supplement: Supplemental data [file jciinsight-9-175462-s107.pdf]

Supplementary information for

Lack of SPNS1 results in accumulation of lysolipids and lysosomal storage disease in mouse models

[1]Hoa T.T. Ha, [1]SiYi Liu, [1]Xuan T.A. Nguyen, [1]Linh K. Vo, [1]Nancy C.P. Leong, [1]Dat T. Nguyen, [1]Shivaranjani Balamurugan, [1,2]Pei Yen Lim, [3]Ya Jun Wu, [4]Eunju Seong, [1]Toan Q. Nguyen, [1,2]Jeongah Oh, [1,2]Markus R. Wenk, [1,2]Amaury Cazenave-Gassiot, [5]Zuhal Yapici, [3]Wei-Yi Ong, [4,6]*Margit Burmeister, [1,2,7,8,9*]Long N. Nguyen

*Corresponding authors: Long N. Nguyen, email: (bchnnl@nus.edu.sg) and *Margit Burmeister, email: margit@umich.edu (For human research).

Supplementary methods

Whole exome sequence analysis of human subjects. Whole exome analysis was performed of all three affected siblings born to consanguineous parents. DNA was isolated from whole blood using the Qiagen Gentra Puregene kit. Next generation exome sequencing was performed by the University of Michigan DNA core facility. Exomes were captured by the SeqCap EZ Exome v3.0 kit (Roche, CA, USA), and paired ends were sequenced on HiSeq2000 to an average depth of 52 X. Variants of interest were validated and tested for segregation patterns by Sanger sequencing. Linkage and Homozygosity Analysis: PLINK was used to identify regions of homozygosity between the three siblings. By linkage and homozygosity mapping, we narrowed the associated genetic region to Chr16: 19.1 Mb – 31.9 Mb (p12.3-p11.2) and Chr16: 55.8 Mb -62.5 Mb (q12.2-q21). In these two regions, exome sequencing identified five genes with homozygous mutations potentially associated with the disease (Table 1). Among them, only the mutations in *SPNS1* and *SEZ6L2,* both on chromosome 16p11.2, have never been reported in homozygous state in *gnomAD* (https://gnomad.broadinstitute.org/). gnomAD is not a control or unaffected database but excludes samples from subjects with pediatric disorders. The mutations in the other three genes have been seen in gnomAD in homozygous state and were therefore excluded. In addition, both *SPNS1* and *SEZ6L2* are highly expressed in the brain with the cerebellum marking the highest level (GTeXportal) and hence are potentially novel candidate genes for the neurological phenotypes including cerebellar hypoplasia in this family. Bioinformatic analyses, including damage potential and conservation scores point to the mutation in *SPNS1*, c.C884T:p.P295L rs532305655, as the most likely candidate, because it is predicted to be disease-causing by the majority of predication programs, of which SIFT is listed in Table 1 as just one example, but is extremely rare – there have been two heterozygous (and no homozygous) alleles reported in Europeans in gnomAD, for an overall allele frequency of about 2 per Million, or 0.000002030.

Antibodies. Primary antibodies including LAMP1 (sc-20011) and GAPDH (sc-32233) were from Santa Cruz Biotechnology. Calreticulin (12238), LC3B (3868S), Cathepsin B (31718S), VDAC (4866S), ATF4 (11815S), p-AKT (4060S), and AKT (9272S) antibodies were from Cell Signaling Technologies. LAMP1 (ab25245) - for IF, GLUT1 (ab40084), LIMP-II (ab176317), and NPC1 (ab134113) antibodies were from Abcam. NPC2 (HPA000835) antibody was from Sigma. Mac-2 (125401) antibody was from Biolegend. MRPS35 (16457) and HMOX1 (10701) were from ProteinTech. OPA1 (612606) was from BD Biosciences. Secondary antibodies including Donkey anti-rabbit 800 (926-32213), Donkey anti-rabbit 680 (926-68023), and Goat anti-mouse 680 (926-68020) antibodies were from Licor company. Secondary antibodies including goat anti-mouse (Alexa Fluor 488 (A28175), goat anti-rabbit (Alexa Fluor 488 (A11034), Alexa Fluor 633 (A21071)), and goat anti-rat (Alexa Fluor 488 (A11006)) antibodies

were from Invitrogen. Antibodies for human and mouse SPNS1 were generated in-house using C-terminal epitope DDRIVVPQRGRSTRVPV.

Sphingosine uptake assay. For whole-cell sphingosine (Sph) uptake assays, the WT and *Spns1*-KO CHO or HEK293 cells were incubated in the transport buffer containing 5 µM [3-^{3}H]-Sph for 15 mins to 4 hours. Transport buffer contains 0.54 mM KCl, 1.3 mM $CaCl_2$, 0.53 mM $MgCl_2$, 0.4 mM $MgSO_4$, 0.37 mM KH_2PO_4, 138 mM NaCl, 0.28 mM Na_2HPO_4, 5.5 mM glycine, 5.6 mM D-glucose, adjusted with HCl to pH 7.5. The cells were washed once with plain DMEM medium containing 0.5% BSA and lyzed in 500µl RIPA buffer for scintillation quantification.

LPC uptake assay. The HEK293 cells were transfected with empty vector (piRES2-EGFP, Addgene) or vectors containing human or mouse SPNS1 with desired mutations using Lipofectamine 2000 (Thermofisher). Mutagenesis was produced by joining two fragments having overlapping ends [1]. After transfection for 24 hours, the transfected cells were incubated with 10 µM [^{14}C]-LPC 18:1 in buffer containing 25 mM sodium citrate, pH 5.5, 150 mM NaCl, 5 mM glucose, 1 mM $MgCl_2$. After 20 mins, the cells were collected and washed twice with DMEM medium containing 0.5% BSA. Then, the cells were lyzed in 400µl RIPA buffer for scintillation quantification.

Genotyping. The following primers were used for genotyping the global *Spns1* knockout: 5'-GGTAGAGCCAGGTGTGTTGGC-3' (forward primer), 5'-GATCTAACCCACCTCCTTCCTTTCC-3' (reverse primer 1), 5'-GGGCAAGAACATAAAGTGACCCTCC-3' (reverse primer 2). For genotyping *Spns1*$^{f/f}$ mice, the following primers were used: 5'-GTAGAGTGGGCAGGGTAATGTG-3' (forward primer F1), 5'-GGATGGTGCGACATCAGTGA-3' (reverse primer R1), that generated an WT band with 448 bp and a floxed band with 482 bp. Deletion of *Spns1* produced a 135 bp band. For genotyping the Cre-ER^{T2}, these primers were used: 5'-AAGGGAGCTGCAGTGGAGTA-3' (common forward primer), 5'-CGGTTATTCAACTTGCACCA-3' (mutant reverse primer), 5'-CCGAAAATCTGTGGGAAGTC-3' (wild-type reverse primer), that generated a positive Cre band of 450 bp (mutant) and an WT band of 297 bp.

Immunostaining of SPNS1 in HEK293 cells. The HEK293 cells were seeded on cover slips inside 24-well plates. Next day, the cells were co-transfected with piRES2 vectors containing human SPNS1 with desired mutations and pLAMP1-mCherry using Lipofectamine 2000 (Thermofisher). 24 hours after transfection, the cells were wash once with PBS, permeabilized with PBS and 0.5% Triton X-100 for 30mins, following by blocking in PBS and 0.5% Triton X-100 and 2.5% normal goat serum (NGS) for 1 hour. After that, the cells were incubated with SPNS1 primary antibody in blocking buffer at 4°C overnight. Next day, the cells were wash twice in PBS and then secondary antibodies goat-anti rabbit 633 for 1 hour. After washing twice with PBS, the cells were co-stain with Hoechst 33342 (1:1000) for 10 mins. Finally, the cells on cover slips were mounted and used for imaging by Zeiss LSM710 confocal microscope.

Immunostaining of mouse liver sections. For immunostaining of the liver sections from adult mice, 10 µm frozen sections were washed in warm PBS for 30 minutes twice to remove OCT completely. Permeabilization was then performed in 0.5% Triton X-100 in PBS (PBST) for 1 hour, followed by blocking in 5% NGS in PBST. Sections were incubated with anti-Cathepsin B (Cell Signaling, 31718S, 1:200), LAMP1 (Abcam, ab25245) or anti-Mac 2 (Biolegend, 125401, 1:250) in blocking buffer at 4°C overnight. All sections were washed three times in PBS at 5 minutes' interval. Depending on different combinations, the sections were incubated with dilution 1:500 of secondary antibodies goat-anti rabbit (A488 or 633), goat anti-rat (A488) in blocking buffer at room temperature. All slides were then washed thrice in PBS before counterstained in Hoechst 33342 (1:1000) for 10 minutes. Goat anti-rabbit 488, goat anti-rabbit 633, goat anti-rat 488 were from Invitrogen. Slides were washed in PBS and then distilled water before mounted in mounting media for imaging by Zeiss LSM710 confocal microscope.

Immunofluorescence quantifications. For quantification of Cathepsin B intensity from immunostaining, four to six images per liver section were captured from different areas. The Cathepsin B mean fluorescence intensity (from Alexa fluor 488 signal) per image was measured using the same threshold by Fiji. Welch's t-test was performed from 15 images for control and 18 images for g*Spns1*-cKO from three mice per genotype. For macrophages' analysis, five to eight images per liver section were captured from different areas. The total number of cells per image was counted from Hoechst signal using Cell Profiler. Mac-2+ cells were indicated using Fiji, and the percentage of these cells was calculated per total cell number in each image. Around 3000 cells from each group were counted. Welch's t-test was performed on 17 images for control and 20 images for g*Spns1*-cKO from three mice per genotype.

To quantify blood vessel density, embryonic brain sections (n=4 per genotype) were stained with GLUT1 antibody. The vascular density in the ganglionic eminence region was determined by Fiji. The immunofluorescence intensity of GLUT1 was quantified by Fiji. The diameters of cortex regions were also measured by Fiji (denoted by the line in Figure 1A).

H&E staining. Embryonic brain and adult liver sections from controls and g*Spns1*-cKO mice were prepared using a microtome and mounted onto microscope slides for overnight drying. Whole embryos collected at E13.5 and liver tissues collected from g*Spns1*-cKO and control mice were fixed in 4% PFA in phosphate buffer saline (PBS) at 4°C overnight. The next day, tissues were submerged in 15% sucrose for 24 hours and then 30% sucrose for 24 hours. The tissues were processed in a Leica tissue processor and then embedded in wax. Tissue sections were prepared using a microtome with 11-15 µm thickness. Serial sections were stained with hematoxylin and eosin. The slides were mounted in D.P.X mounting media and covered with coverslips. The sections were imaged using a Tissue scanner (FAXS) or Olympus microscope.

Western blot. To prepare the samples for WB analysis, embryos and tissues were homogenized in lysis buffer (with a ratio of 2-3 µl per 1 mg wet tissue) containing 150 mM NaCl, 1% Triton X-100, 10 mM Tris-HCl pH 8.0 with protease inhibitor (Invitrogen) and phosphatase inhibitor (Sigma) using FastPrep-24 MP with beads for 2 minutes in the cold room. Mammalian cells from HEK293 and CHO cell cultures were also lyzed in the lysis buffer for 30 minutes using a rotary shaker at 50 rpm in the cold room. Protein concentrations of lysates were measured using the BCA method. The same amounts of total proteins were added with Laemmli buffer and resolved in 10-12% SDS-PAGE at 100 V for 100 minutes. Proteins were transferred to nitrocellulose membranes at 20 V in the cold room overnight. Membranes were then blocked in TBST (Tris-buffered saline with 0.1% Tween 20) containing 5% skim milk at room temperature for 1 hour. Primary antibodies diluted as advised by the manufacturer's instruction in the same blocking buffer were incubated for 2 hours at room temperature or were incubated with the membranes in the cold room overnight. Next, the membranes were washed with TBST three times (5-minute intervals) before incubation in secondary antibodies (1:10,000) for 1 hour at room temperature. Membranes were then washed three times in TBST again before being visualized with ChemiDoc (Biorad).

RNA-sequencing analysis. Liver tissues from control and g*Spns1*-cKO mice were harvested and RNA was isolated using RNeasy kit (QIAgen, cat. 74104). RNA-sequencing and sample analysis was done by NovogeneAIT (Singapore). The raw data and analysis were uploaded to Gene Expression Omnibus (GEO) and the accession number is GSE240323.

Enrichment of lysosomes from liver tissues. Control and g*Spns1*-cKO mice were injected with 4 µl/g body weight of a 17% (w/v in 0.9% NaCl) Triton WR1339 solution (Sigma Aldrich) for three days prior to fractionation of lysosomes. The non-perfused livers were homogenized in five volumes (usually 5 ml) of ice-cold 0.25 M sucrose with three strokes in a 10 ml Potter-Elvehjem homogenizer. The homogenates were centrifuged at 1000 ×g for 10 minutes at 4°C. An amount of 4 ml supernatant was collected into 15 ml tubes, and the remaining pellet was resuspended in 3.5 ml 0.25 M sucrose, followed by another step of centrifugation at 1000 ×g

for 10 minutes at 4°C for the second collection of 4 ml supernatant. Both supernatants (post-nuclear supernatant, PNS) were pooled and transferred to ultracentrifuge tubes. The supernatant was filled with 0.25 M sucrose up to 9 ml and centrifuged in an ultracentrifuge at 56,000 ×g (P40ST Swing rotor, Hitachi) for 7 minutes at 4°C. The supernatant was discarded. After resuspension of the pellet with 9 ml 0.25 M sucrose, followed by another step of ultracentrifugation at 56,000 ×g (P40ST Swing rotor, Hitachi) for 7 minutes at 4°C, the supernatant was also discarded. The pellet was resuspended in sucrose solution (3.5 ml) with a density of p=1.21 (resulting in the mitochondrial-lysosomal fraction (ML fraction). The ML fraction was transferred to an ultracentrifuge tube and overlaid by sequential steps with 2.25 ml of sucrose solutions of p=1.15, 2.25 ml of p=1.14, and 1 ml of p=1.06 yielding a discontinuous sucrose gradient. The discontinuous gradient was centrifuged at 110,000 ×g in a swinging bucket rotor (P40ST Swing rotor, Hitachi) for 150 minutes at 4°C. Lysosome fractions were collected at the interphase between p=1.14 and 1.06 sucrose (F2-fraction) after removing the top layer (F1 fraction).

Internal standards for lipidomic analysis. Internal standards (IS) solutions were prepared in butanol/methanol (BuMe) (1:1, v/v). The composition and concentration of the solutions were adjusted for the different sample types and analytical methods. For sphingolipid analysis, the internal standards Cer/Sph II from Avanti Polar Lipids (Cat LM 6005) were used. The IS in the sphingolipid extraction solvent consisted of 71.7 nM SM d18:1/12:0, 70.2 nM Sph d17:1, 71.4 nM Sphingosine d17:0, 71.1 nM Ceramide d18:1/12:0, 69.9 nM Hexosylceramide d18:1/12:0, 72 nM Lactosylceramide d18:1/12:0. For phospholipid analysis, the phospholipid IS for embryonic brain samples were used as follows: 1.18 µM PE (17:0/17:0), 3.12 µM PC 26:0 (13:0/13:0), 0.13 µM LPC 20:0, 0.165 µM LPE 14:0, 0.34 µM PI 25:0, all of which were purchased from Avanti. The phospholipid IS for adult liver included 1.6 nM d3-Acyl Carnitine 16:0, 0.36 µM LPC 20:0, 5.57 µM PC 26:0 (13:0/13:0), 0.77 µM LPE 14:0, 3.01 µM PE (17:0/17:0), 0.75 µM DMPS, 0.83 µM PI 25:0, 0.90 µM SM 12:0 (d18:1/12:0). The phospholipid IS for lysosome fraction included SPLASH Mix (Avanti, 330707).

Methods for lipid extractions

Embryonic brain and liver tissues: Embryonic brain tissues at E13.5 and PBS-perfused liver tissues from g*Spns1*-cKO and control mice were collected and immediately transferred to dry ice before storage at -80ºC. Tissue samples were then homogenized using FastPrep-24 MP with beads in 150 mM ammonium bicarbonate buffer, pH 7.8, with a ratio of 10 µl buffer per 1 mg wet tissue for 2 minutes in a cold room (4°C). The protein concentration of samples was measured using the BCA method. For lipid extraction, 200 µl of butanol/methanol (1:1) solution containing appropriate standards (phospholipid or sphingolipids, see above) were added to 20 µl of tissue homogenate. The IS-spiked samples were then sonicated in a water bath for 30 minutes, followed by centrifugation at 14,000 ×g for 10 minutes. The supernatant was then transferred to mass spectrometry vials for LC-MS/MS analysis.

HEK293 and CHO cell: Cell samples were grown in 6-well plates, either in growth medium (DMEM with 10% fetal bovine serum, FBS) or starved under serum-deprived medium (DMEM without amino acids and FBS (Wako, 048-33575)) for 4 hours. Cells were harvested with cell scrappers using 1 ml PBS per well, then centrifuged at 3,000 ×g for 5 minutes to collect the cell pellets. An amount of 200 µl butanol/methanol (1:1) solution containing internal sphingolipid standard mix or phospholipid standard mix (see above) was added to the cell pellets. The IS spiked samples were then sonicated in a water bath for 30 minutes, followed by centrifugation at 14,000 ×g for 10 minutes. The supernatant was then transferred to mass spectrometry vials for LC-MS/MS analysis. The pellet was dried in Speedvac and then solubilized in 200 µl RIPA to measure protein concentration using BCA method for normalization.

Lysosome fractions: To extract sphingolipids from lysosomal fractions extracted from livers of control and *gSpns1*-cKO mice, 10 µl of each lysosomal fraction was used for lipid extraction using the above-mentioned procedure (addition of 200 µl butanol/methanol (1:1) containing the phospholipid internal standards). To extract phospholipids from lysosomal fraction, the knockout samples were first diluted 10 times in the same sucrose buffer (p=1.06),

and then 20 µl of the lysosomal fraction from control and KO samples was used for lipid extraction as as described above (addition of 200 µl BuMe containing phospholipid internal standards, Splash Mix, Avanti). Protein concentrations in the lysosomal fractions were measured by the BCA method for normalization. Normalization factor was calculated based on the protein concentrations, but then separately between control and *gSpns1*-cKO liver (see calculation in Supplemental S14-15. After normalization with IS, measured lipids from lysosome fractions were further normalized to normalization factors.

Lipids quantification by LC/MS/MS

Samples were randomized before extraction and analysis. Blank samples (empty tubes for cell samples and 10 µL MilliQ water for other sample types), matrix blanks (samples extracted without spiking internal standards), and pooled QC samples were used to assess method performance. In addition, diluted pooled QC samples were used to assess response linearity. Blanks, blank extracts, QC, and diluted QC were interspersed with study samples throughout the analytical run.

Sphingolipids were separated using a reverse phase column (Agilent RRHD Eclipse Plus column, 959758-902, C18, 2.1x100 mm, 1.8 µm) on an Agilent 6495A2 Triple Quadrupole mass spectrometer (Agilent Technologies). Mobile phases A (60% Methanol (Thermo Fisher Scientific Inc.), 40% MilliQ water, 10 mM Ammonium Acetate (Sigma-Aldrich), 0.2% formic acid (Sigma-Aldrich)) and B (60% Methanol (Thermo Fisher Scientific Inc.), 40% Isopropanol (Thermo Fisher Scientific Inc.), 10 mM Ammonium Acetate (Sigma-Aldrich), 0.2% formic acid (Sigma-Aldrich)) were mixed using the following gradient: 0–3 minutes, 0-10% B; 3-5 minutes, 10-40% B; 5-5.3 minutes, 40-55% B; 5.3-8 minutes, 55-60% B, 8-8.5 minutes, 60-80% B; 8.5-10.5 minutes, 80% B; 10.5-16 minutes, 80-90% B; 16-19 minutes, 90% B, 19-22 minutes, 90-100% B. The flow rate was 0.4 ml/min, and the sample injection volume was 2 µl.

Phospholipids were separated using a HILIC column (Kinetex 2.6 µm HILIC 100 Å, 150 × 2.1 mm, Phenomenex, Torrance, CA, USA) on an Agilent 6495A2 (Agilent Technologies). Mobile phases A (50% acetonitrile (LC-MS grade, Thermo Fisher Scientific Inc.) 50% 25 mM ammonium formate (Sigma-Aldrich) pH = 4.6) and B (95% acetonitrile and 5% 25 mM ammonium formate pH = 4.6) were mixed at the following gradient: 0–6 minutes, 99–75% B; 6–7 minutes, 75-10% B; 7–7.1 minutes, 10–99.9% B; 7.1–10.1 minutes, 99.9% B. The flow rate was 0.5 ml/minute, and the sample injection volume was 0.5 µL for lysosome fraction and 0.25 µL for liver samples.

The MS parameters on Agilent 6495A2 were as follows: electrospray ionization, gas temperature 200°C, gas flow 15 l/minute, sheath gas flow 12 l/minute, and capillary voltage 3,500 V. Sphingolipids and phospholipids were quantified at the sum composition level using multiple reaction monitoring (MRM).

Data Processing for lipidomic. The raw data was processed using Agilent MassHunter Quantitative Analysis software for QQQ version B.08 (Agilent Technologies, Santa Clara, CA, USA). The areas under the curve (AUC) were manually inspected and checked for retention time. Blanks and QC samples were used to assess the following parameters for each MRM transition: coefficient of variation (CV) in QCs, signal in blanks, and linearity in diluted QC samples. Only MRM transitions satisfying the established criteria were used for quantification: CV in QCs < 25%, signal QC/blank > 10, and Pearson R2 in diluted QC > 0.8. Isotopic correction of signals based on the precursor ions was performed using LICAR [2] for HILIC analysis. Finally, the lipid samples were normalized to relevant internal standards and protein concentration.

Metabolomics

For metabolomics, we collected 50-100 mg tissues from PBS perfused livers of *Spns1*$^{f/f}$-Rosa26CreERT^2 (g*Spns1*-cKO) and control mice. The untargeted metabolomics was performed by Metabolon. Detail methods for metabolomic analysis from Metabolon is described below.

For sample preparation: Liver samples were prepared using the automated MicroLab STAR® system from Hamilton Company. Several recovery standards were added prior to the

first step in the extraction process for QC purposes. To remove protein, dissociate small molecules bound to protein or trapped in the precipitated protein matrix, and to recover chemically diverse metabolites, proteins were precipitated with methanol under vigorous shaking for 2 min (Glen Mills GenoGrinder 2000) followed by centrifugation. The resulting extract was divided into five fractions: two for analysis by two separate reverse phases (RP)/UPLC-MS/MS methods with positive ion mode electrospray ionization (ESI), one for analysis by RP/UPLC-MS/MS with negative ion mode ESI, one for analysis by HILIC/UPLC-MS/MS with negative ion mode ESI, and one sample was reserved for backup. Samples were placed briefly on a TurboVap® (Zymark) to remove the organic solvent. The sample extracts were stored overnight under nitrogen before preparation for analysis. For metabolite analysis, ultrahigh Performance Liquid Chromatography-Tandem Mass Spectroscopy (UPLC-MS/MS) was employed. All methods utilized a Waters ACQUITY ultra-performance liquid chromatography (UPLC) and a Thermo Scientific Q-Exactive high resolution/accurate mass spectrometer interfaced with a heated electrospray ionization (HESI-II) source and Orbitrap mass analyzer operated at 35,000 mass resolution. Prior to analysis, the sample extract was dried then reconstituted in solvents compatible to each of the four methods. Each reconstitution solvent contained a series of standards at fixed concentrations to ensure injection and chromatographic consistency. One aliquot was analyzed using acidic positive ion conditions, chromatographically optimized for more hydrophilic compounds. In this method, the extract was gradient eluted from a C18 column (Waters UPLC BEH C18-2.1x100 mm, 1.7 µm) using water and methanol, containing 0.05% perfluoropentanoic acid (PFPA) and 0.1% formic acid (FA). Another aliquot was also analyzed using acidic positive ion conditions; however, it was chromatographically optimized for more hydrophobic compounds. In this method, the extract was gradient eluted from the same afore mentioned C18 column using methanol, acetonitrile, water, 0.05% PFPA and 0.01% FA and was operated at an overall higher organic content. Another aliquot was analyzed using basic negative ion optimized conditions using a separate dedicated C18 column. The basic extracts were gradient eluted from the column using methanol and water, however with 6.5mM Ammonium Bicarbonate at pH 8. The fourth aliquot was analyzed via negative ionization following elution from a HILIC column (Waters UPLC BEH Amide 2.1 x 150 mm, 1.7 µm) using a gradient consisting of water and acetonitrile with 10mM Ammonium Formate, pH 10.8. The MS analysis alternated between MS and data-dependent MS^n scans using dynamic exclusion. The scan range varied slighted between methods but covered 70-1000 m/z. Raw data files are archived and extracted as described below.

Data Extraction and Compound Identification from metabolomics. Raw data was extracted, peak-identified and QC processed using Metabolon's hardware and software. These systems are built on a web-service platform utilizing Microsoft's .NET technologies, which run on high-performance application servers and fiber-channel storage arrays in clusters to provide active failover and load-balancing. Compounds were identified by comparison to library entries of purified standards or recurrent unknown entities. Metabolon maintains a library based on authenticated standards that contains the retention time/index (RI), mass to charge ratio (*m/z)*, and chromatographic data (including MS/MS spectral data) on all molecules present in the library. Furthermore, biochemical identifications are based on three criteria: retention index within a narrow RI window of the proposed identification, accurate mass match to the library +/- 10 ppm, and the MS/MS forward and reverse scores between the experimental data and authentic standards. The MS/MS scores are based on a comparison of the ions present in the experimental spectrum to the ions present in the library spectrum. While there may be similarities between these molecules based on one of these factors, the use of all three data points can be utilized to distinguish and differentiate biochemicals. More than 3300 commercially available purified standard compounds have been acquired and registered into LIMS for analysis on all platforms for determination of their analytical characteristics. Additional mass spectral entries have been created for structurally unnamed biochemicals, which have been identified by virtue of their recurrent nature (both

chromatographic and mass spectral). These compounds have the potential to be identified by future acquisition of a matching purified standard or by classical structural analysis.

Metabolite curation. A variety of curation procedures were carried out to ensure that a high-quality data set was made available for statistical analysis and data interpretation. The QC and curation processes were designed to ensure accurate and consistent identification of true chemical entities, and to remove those representing system artifacts, mis-assignments, and background noise. Metabolon data analysts use proprietary visualization and interpretation software to confirm the consistency of peak identification among the various samples. Library matches for each compound were checked for each sample and corrected if necessary.

Metabolite Quantification and Data Normalization. Peaks were quantified using area-under-the-curve. For studies spanning multiple days, a data normalization step was performed to correct variation resulting from instrument inter-day tuning differences. Essentially, each compound was corrected in run-day blocks by registering the medians to equal one (1.00) and normalizing each data point proportionately (termed the “block correction”; Figure 2). For studies that did not require more than one day of analysis, no normalization is necessary, other than for purposes of data visualization. In certain instances, biochemical data may have been normalized to an additional factor (e.g., cell counts, total protein as determined by Bradford assay, osmolality, etc.) to account for differences in metabolite levels due to differences in the amount of material present in each sample.

Description of three patients with P295L mutation.

The affected subjects consist of 3 siblings from 4 children born from parents' first-degree cousin marriages had neurodevelopmental problems and neurological, especially cerebellar, impairment. The parents and one sibling were unaffected. Subjects and parents gave informed consent for the genetic study of neurological disorders, which was approved by the University of Michigan IRB.

Case 1: Motor and mental developmental delay since birth. Able to walk, though unsteadily, by age 3, but even by age 16 and 20 can’t run. First words age 3-4. Able to read and write by age 16. Irregular menstruation and ovarian cysts. At that age 16, mildly dysarthic but understandable speech, limited intellectual capacity, hence in special education. Cerebellar tests such as Romberg sign and tandem walking mildly impaired. No tremor or titubation.

Case 2: Able to walk by age 2, and run by age 5. Started speaking by age 2. Academically, severely affected, being able to learn to read and write at age 15-16. On neurological examination, cerebellar tests show mild to moderate impairment: dysarthric but understandable speech, Romberg (-), but cannot do tandem walking. Intentional tremor and titubation are present. Ataxic features though mild are noticeable. Speech has improved over time.

Case 3: Able to walk, albeit unsteadily, by age 2. Could not run, and fell easily. Speaking started by age 4, learned to read and write at age 9. By age 9, speech was dysarthric but has improved. Cerebellar tests showed impairment: no tandem walking, and intention tremor was evident. However, by age 12, coordination is improved, can run and climb stairs well, plays football, and has better academic level than siblings.

References

1. Ho SN, et al. Site-directed mutagenesis by overlap extension using the polymerase chain reaction. Gene 1989;**77**(1):51-59.
2. Gao L, et al. LICAR: An Application for Isotopic Correction of Targeted Lipidomic Data Acquired with Class-Based Chromatographic Separations Using Multiple Reaction Monitoring. Analytical Chemistry 2021;**93**(6):3163-71 doi: 10.1021/acs.analchem.0c04565.

Supplemental table 1: **Bioinformatic analysis of five mutations in the linkage interval found homozygous mutated in the three children with developmental delay and neurological disorder in the family ascertained.**

Indications speaking again a mutation/gene are italicized, evidence in favor is bolded.

Gene name	*DNAH3*	*SPNS1*	*SEZ6L2*	*INO80E*	*SLC12A3*
Chr. 16location	21042438	28993296	29908260	30016644	56906568
Variant	c.A5368T:p.I1790F	c.C884T:p.P295L	c.C262T:p.P88S	c.C616A:p.P206T	c.C965T:p.A322V
SNP	rs148202152	rs532305655	rs201397581	rs201487520	rs142679083
Frequency (gnomAD)	*5269; 16 homozygotes*	**1; 0 homozygotes**	**3; 0 homozygotes**	*842; 4 homozygotes*	*3888; 11 homozygotes*
Expression in cerebellum	*no*	**yes**	**yes**	**yes**	*no*
SIFT predict	**Deleterious**	**Deleterious**	*Tolerated*	*Tolerated*	*Tolerated*
CADD score	**5.99**	**7.47**	*1.12*	*0.27*	*1.76*
REVEL score	*0.334*	**0.808**	*0.016*	*0.052*	*0.28*

SNP- single nucleotide polymorphism; rs number is listed

gnomAD – The genome aggregation database

SIFT – sorting intolerant from tolerant

CADD – combined annotation dependent depletion – higher score means variant less tolerated

REVEL – rare exome variant ensemble learner – higher score means variant less tolerated

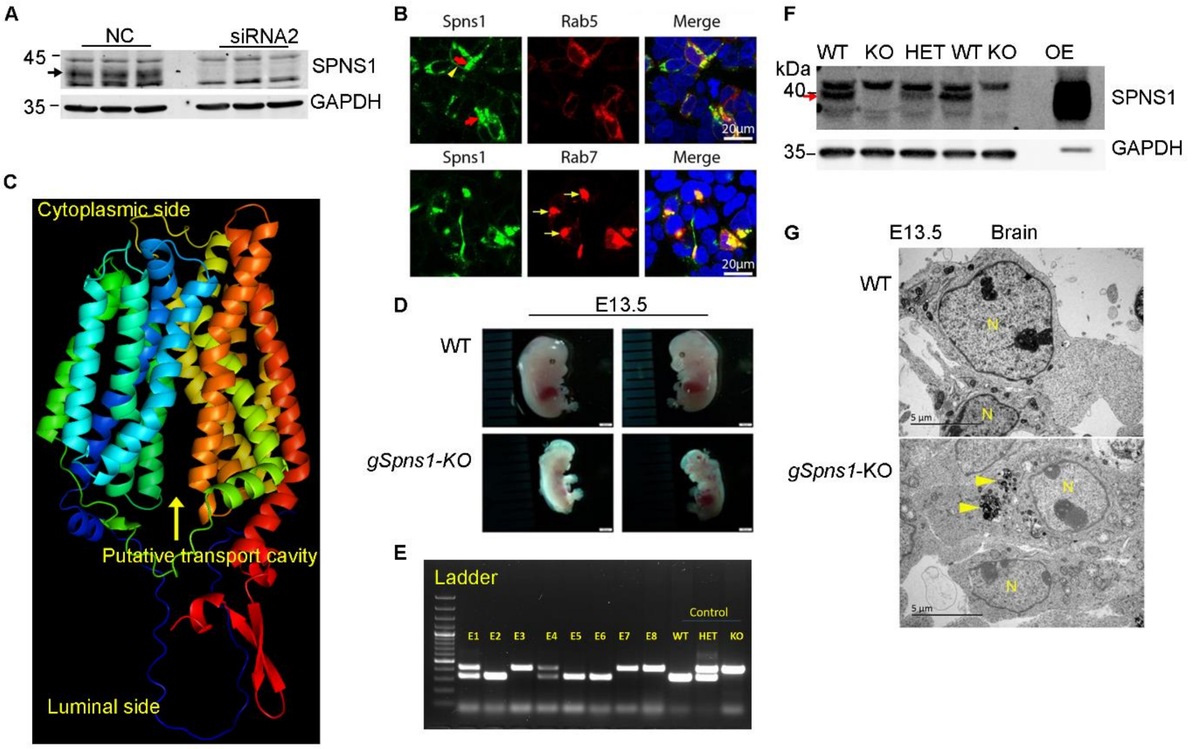

***Figure S1. SPNS1 is a putative lysosomal transporter required for embryonic development**. **A**. Validation of polyclonal antibodies for SPNS1 in CHO cells. Expression of SPNS1 in wild-type and Spns1 knockdown CHO cells using siRNAs. The data show that endogenous SPNS1 is expressed in CHO cells with a molecular weight of approximately 40 kDa. **B**, SPNS1 is co-expressed with Rab7-RFP or Rab5-RFP in HEK293 cells. SPNS1 is co-localized with late endosomal maker Rab7 (lower panel, arrows), but not with early endosomal marker Rab5 (upper panel, arrows). SPNS1 is present in or near plasma membrane (upper panel, arrowhead). **C**, Modelled structure of human Spns1 from AlphaFold2. Arrow shows the putative transport cavity predicted for a solute transporter. **D**, Whole-body deletion of Spns1 results in early lethality in mice. Shown are representative images from 1 WT and 1 KO embryos of the same litter. The whole body of Spns1 knockout is smaller with severe brain developmental defects. **E**, PCR genotyping of embryos from a crossing of two heterozygous mice. WT: wild-type, HET: heterozygous, KO: knockout of Spns1. **F**, Western blot analysis of SPNS1 from the embryos. OE: overexpression of Spns1 in HEK293 cells as a positive control. **G**, Representative electron microscopic images of brain sections from E13.5 WT and Spns1 knockout embryos. Knockout embryos had an accumulation of condensed membranous materials in the cytoplasm (arrowheads). N, nuclei. n=3 per genotype.*

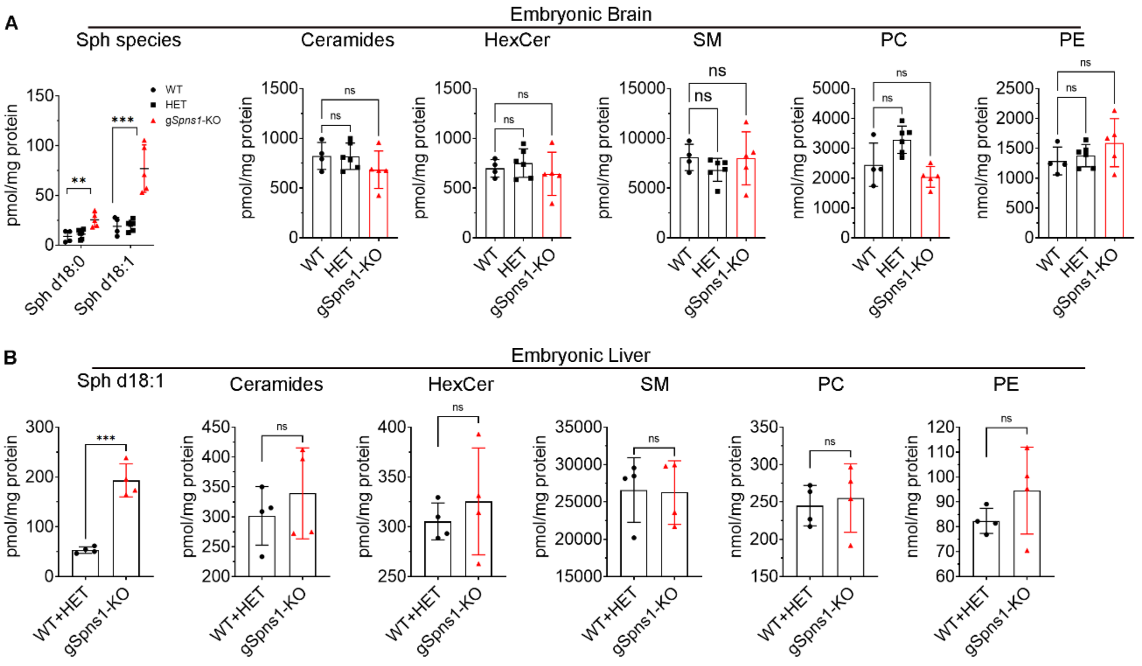

***Figure S2. Deletion of Spns1 causes sphingosine accumulation in both embryonic brains and livers. A-B**, Lipidomic analysis of embryonic brains and embryonic livers at E13.5. WT: wild-type, HET: heterozygous, KO: knockout. HexCer: hexosylceramides, SM: sphingomyelins, sph: sphingosine, PC: phosphatidylcholines, PE: Phosphatidylethanolamine. **p<0.01; ***p<0.001; ****p<0.0001; ns, not significant. Data are expressed as mean ± SD. All statistical significance was determined by two-sided unpaired t-test.*

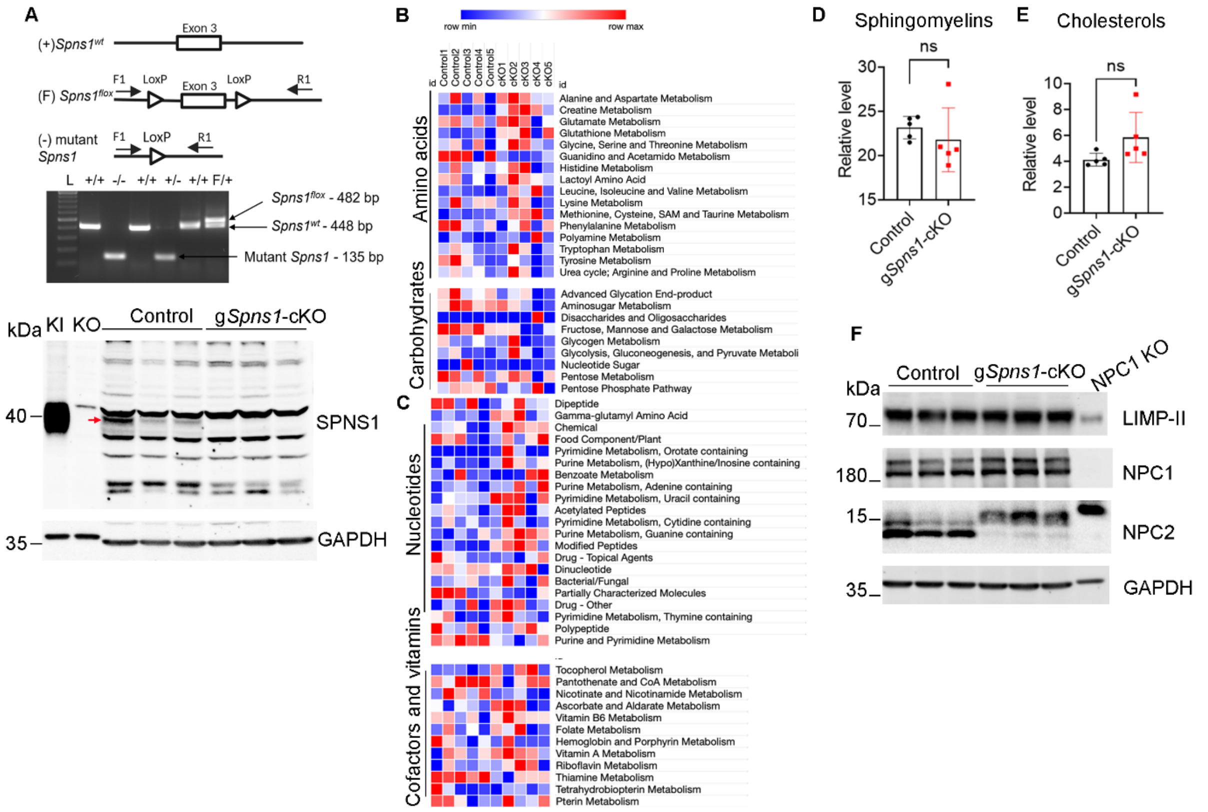

Figure S3. Generation and metabolomic analysis of conditional knockout of Spns1. A*, Generation of conditional knockout of Spns1 and validation of Spns1 deletion in livers of control (Spns1$^{f/f}$) and gSpns1-cKO mice (Spns1$^{f/f}$; ROSA26-CreER^{T2} mice) after induction with tamoxifen using western blot. Arrow shows the Spns1 protein band which was reduced in the knockouts. n=3 per genotype.* ***B-C****, Metabolomic analysis of amino acids, carbohydrates, nucleotides, and vitamins and cofactors from livers of control and gSpns1-cKO mice.* ***D-E****, Metabolomic analysis of sphingomyelins and cholesterols in the livers of control and gSpns1-cKO liver. ns, not significant. Data are expressed as mean ± SD. All statistical significance was determined by two-sided unpaired t-test.* ***F****, Western blot analysis of lysosomal proteins including LIMP-II, NPC1, and NPC2 in livers of control and gSpns1-cKO mice. n=3 per genotype.*

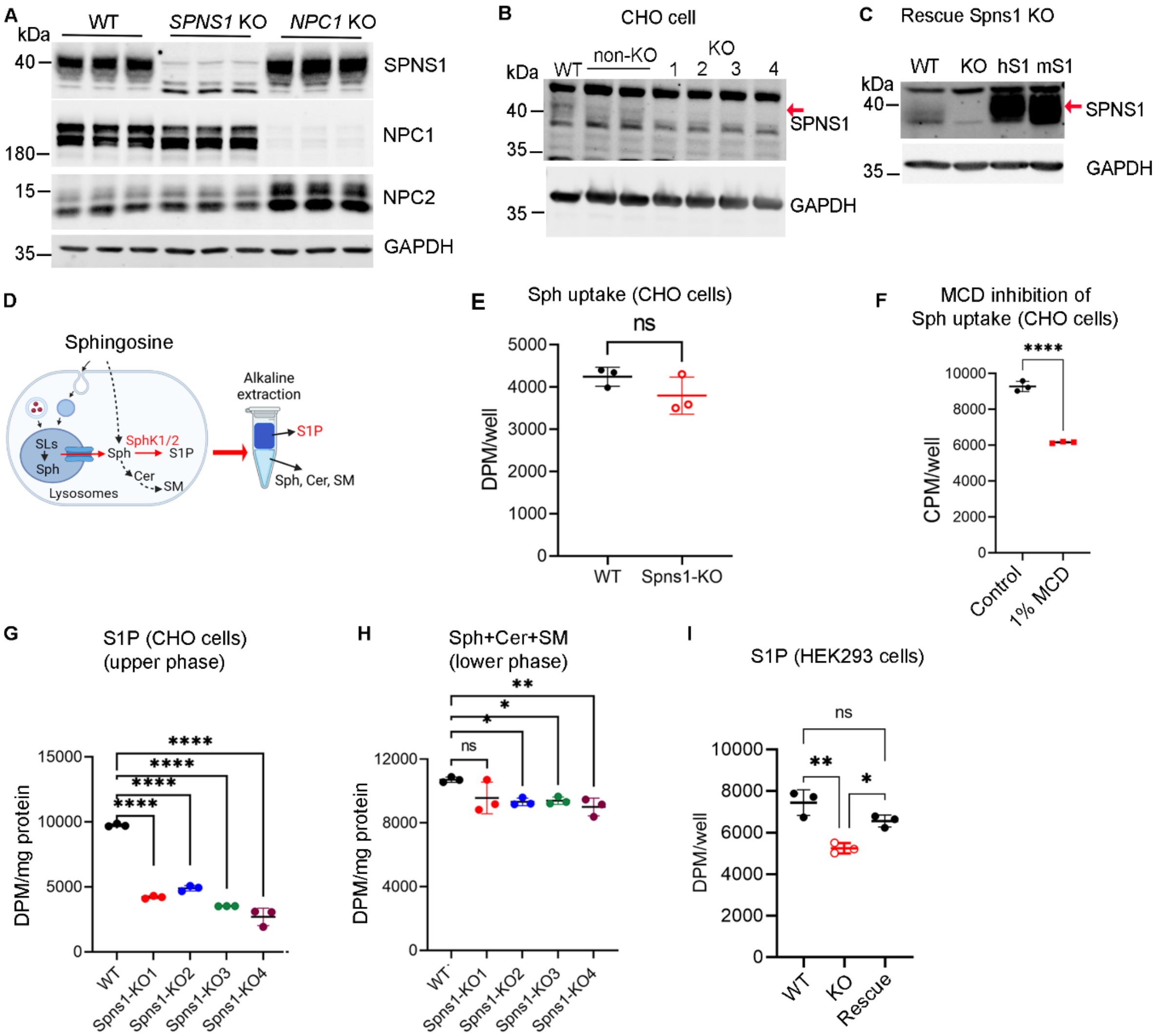

Figure S4. SPNS1 is required for sphingosine and LPC release from lysosomes. A-C, *Generation of Spns1 knockout and knock-in cells using CRISPR/Cas9 technology. Western blot analysis of SPNS1, NPC1, and NPC2 proteins from the indicated knockout lines (in A). Western blot analysis of SPNS1 protein from WT and KO CHO cells (in B) and with rescue clones (in C). Arrow shows the Spns1 protein band which is reduced in the knockout cell lines. WT: wild-type, KO: Spns1 knockout, and KI: Spns1 knock-in. hS1: Spns1 KO was rescue with hSPNS1. mS1: Spns1 KO was rescued with mSPNS1.* ***D****, Illustration of [3-^{3}H]-sphingosine transport assays. CHO cells were starved in medium without amino acids and serum for 1 hour. The cells were then added with radioactive sphingosine and continued incubating for 4 hours.* ***E****, Total radioactive signals of [3-^{3}H]-sphingosine after 4-hour incubation in CHO WT and Spns1 KO cells.* ***F****. MCD inhibition of sphingosine uptake in WT CHO cells for 0.5 hours. Each symbol represents one replicate. (two-sided unpaired t-test, n=3, ****p<0.0001. Data are expressed as mean ± SD).* ***G****, Radioactive S1P levels (upper phase) from WT and different Spns1-KO clones from CHO cells.* ***H****, Radioactive sphingolipid levels (lower phase) from WT and different Spns1-KO clones.* ***I-J****, Radioactive S1P levels (upper phase) from WT, Spns1-KO with rescue in CHO cells (**I**) and in HEK293 cells (**J**). Each symbol represents one replicate. one-way ANOVA, n=3, *p<0.05; **p<0.01; ***p<0.001; ns, not significant. Data are expressed as mean ± SD).*

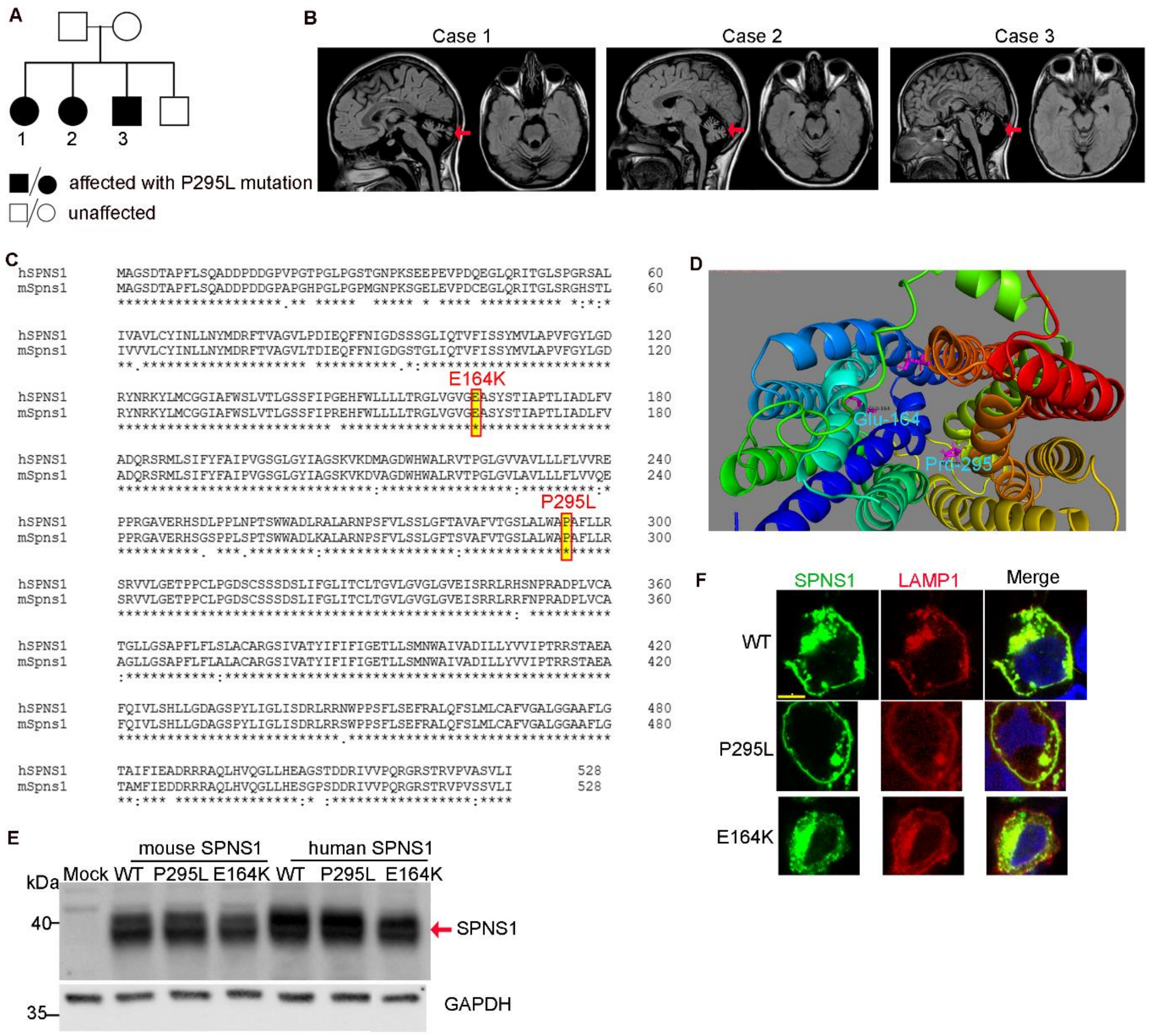

Figure S5. Identification of a missense variant of SPNS1 in 3 human subjects with neurodevelopmental defects. A*, Pedigree of the family with a homozygous variant at P295L of SPNS1. Three siblings with P295L variant exhibited neurodevelopmental problems and ataxia.* ***B****, MRI images of the brain of three siblings showing the cerebellar defect (red arrows). Case 1: 18 years old, case 2: 16 years old, case 3: 9 years old.* ***C****, Alignment of the amino acid sequences of mouse SPNS1 and human SPNS1 shows the conservation of E164 and P295.* ***D****, AlphaFold2 model of human SPNS1 structure with showing the location of E164 and P295.* ***E****, Western blot analysis of SPNS1 expression in HEK293 cells transfected with empty piRES vector (mock), wildtype (WT), P295L, E164K in mouse SPNS1 or human SPNS1, respectively.* ***F****, Immunofluorescence analysis of the localization human SPNS1 and the indicated mutants in HEK293 cells. LAMP1 was used as a lysosomal marker.*

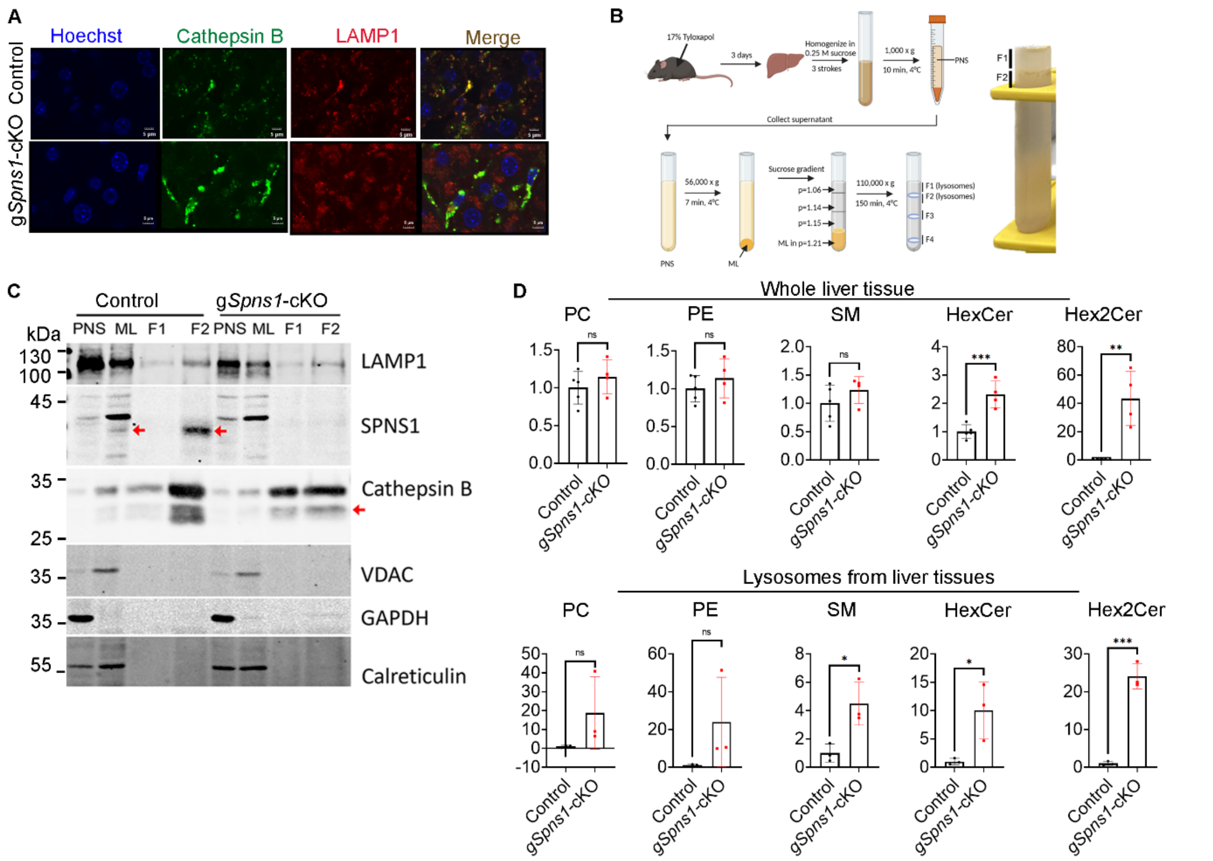

Figure S6. Lack of Spns1 brings about phenotypes reminiscent of lysosomal storage diseases. ***A***, *Representative images of immunofluorescent analysis of Cathepsin B (green) and LAMP1 (red) in control and gSpns1-ckO livers, co-stained with Hoechst. n=3 per group.* ***B***, *Schematic illustration of isolation of lysosomes from mouse liver tissues.* ***C***, *Western blot analysis of lysosomal (LAMP1 and Cathepsin B), mitochondria (VDAC), and endoplasmic reticulum (Calreticulin) markers from liver lysosome fractions.* ***D***, *Lipidomic analysis of whole liver and extracted lysosome (F1 and F2) from livers of control and gSpns1-cKO mice. n=3 per genotype. *p<0.05; **p<0.01; ***p<0.001; ns, not significant, Data are expressed as mean ± SD. All statistical significance was determined by two-sided unpaired t-test.*

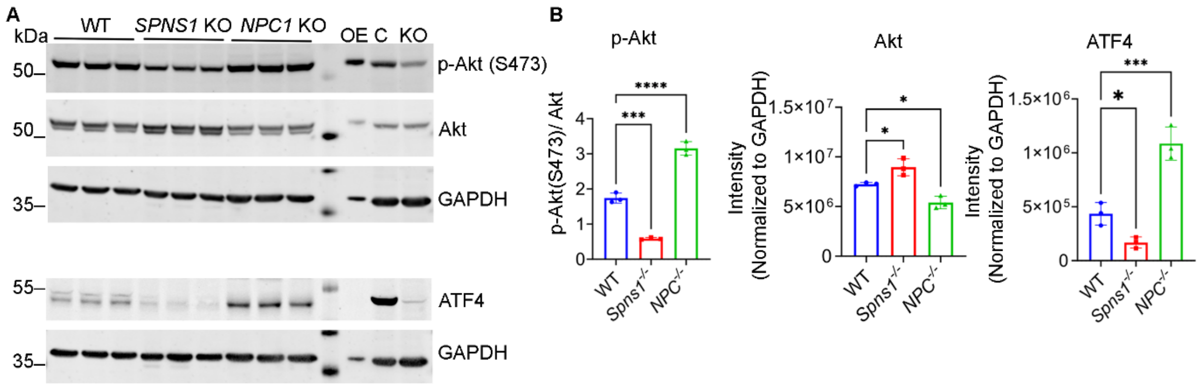

Figure S7. Alteration of ATF4 and p-Akt signaling in SPNS1 KO cells. A, *Western blot analysis of ATF4, p-Akt, and total Akt in wild-type (WT) HEK293 cells, SPNS1 KO cells, and NPC1 KO cells. OE: SPNS1 overexpression in HEK293 cells. C, KO: control and gSpns1-cKO liver samples, respectively.* ***B****, Quantification of expression of the protein bands in* ***A*** *after normalization to GAPDH. **p<0.01; ****p<0.0001; ns, not significant. Data are expressed as mean ± SD. Statistical significance was determined by One-way ANOVA.*

Legends for supplemental data.

Supplemental data S1: Sphingolipids and phospholipid analysis from brains of controls and g*Spns1*-KO embryos.

Supplemental data S2: Sphingolipids and phospholipid analysis from livers of controls and g*Spns1*-KO embryos.

Supplemental data S3: Metabolomic analysis of Lysophospholipids and Sphingolipids from liver tissues of controls and gSpns1-cKOs.

Supplemental data S4: Metabolomic analysis of phospholipids and cholesterol from liver tissues of controls and gSpns1-cKOs.

Supplemental data S5: Metabolomic analysis results for all lipid metabolites from liver tissues of controls and gSpns1-cKOs.

Supplemental data S6: Metabolomic analysis of levels of amino acid metabolites from liver tissues of controls and gSpns1-cKOs.

Supplemental data S7: Metabolomic analysis of levels of carbohydrate metabolites from liver tissues of controls and gSpns1-cKOs.

Supplemental data S8: Metabolomic analysis of levels of nucleotide, vitamin, and cofactor metabolites from liver tissues of controls and gSpns1-cKOs.

Supplemental data S9: Lipidomic analysis of WT and SPNS1-KO in HEK293 cells.

Supplemental data S10: Lipidomic analysis of WT and SPNS1-KO in CHO cells.

Supplemental data S11: Lipidomic analysis of WT, SPNS1-KO, and NPC1 knockout in HEK cells.

Supplemental data S12: Sphingolipid (SL) analysis of whole liver tissues from control and g*Spns1*-cKO mice.

Supplemental data S13: Phospholipid (PL) analysis of whole liver tissues from control and g*Spns1*-cKO mice.

Supplemental data S14: Sphingolipid analysis of lysosome extraction from liver tissues of control and gSpns1-cKO mice.

Supplemental data S15: Phospholipid analysis of lysosome extraction from liver tissues of control and gSpns1-cKO mice.

Supplemental data S16: RNA-sequencing results of whole liver tissues from control and g*Spns1*-cKO mice.
